# Identifying Metabolite–Disease Associations via Messaging in Hypergraphs

**DOI:** 10.3390/metabo16020116

**Published:** 2026-02-09

**Authors:** Fuheng Xiao, Yihao Ran, Zhanchao Li

**Affiliations:** 1 School of Chemistry and Chemical Engineering, Guangdong Pharmaceutical University, Guangzhou 510006, China; 2112240053@stu.gdpu.edu.cn; 2 Guangdong Metabolic Diseases Research Center of Integrated Chinese and Western Medicine, Institute of Traditional Chinese Medicine, Guangdong Pharmaceutical University, Guangzhou 510006, China; 2112450095@stu.gdpu.edu.cn

**Keywords:** metabolites, diseases, hyperedge, machine learning

## Abstract

**Background:** Traditional machine-learning approaches face challenges when attempting to integrate diverse biological information for predicting metabolite–disease relationships. The intricate connections linking metabolites, diseases, proteins, and Gene Ontology (GO) annotations present substantial obstacles for conventional pairwise graph representations, which prove inadequate for modeling such complex multi-way interactions. **Methods:** An innovative hypergraph-based framework (DHG-LGB) was developed to exploit this complexity through conceptualizing diseases as hyperedges. Within this architecture, individual hyperedges link multiple vertices including metabolites, proteins, and GO annotations, thereby enabling richer representation of the biological networks underlying metabolite–disease relationships. Metabolite–disease relationships were encoded as low-dimensional vectors through hypergraph neural network (HGNN) operations incorporating Laplacian smoothing and message propagation mechanisms. LightGBM (LGB) was used to construct a model for identifying the potential metabolite–disease associations. **Results:** Under 5-fold cross-validation, DHG-LGB achieved 98.87% accuracy, 91.77% sensitivity, 99.58% specificity, 95.60% precision, Matthews correlation coefficient (MCC) of 0.9305, receiver operating characteristic area under curve (AUC) of 0.9983, and precision-recall area under curve (AUPRC) of 0.9860. The framework maintained strong performance when tested with varying positive-to-negative ratios (spanning 1:1 through 1:10), consistently achieving AUC values exceeding 0.9954 and AUPRC values above 0.9820, thereby confirming excellent robustness and generalization capability. Comparative evaluations against existing methodologies verified the superiority of DHG-LGB. **Conclusions:** The DHG-LGB framework delivers more comprehensive modeling of biological interactions relative to conventional approaches and substantially enhances predictive accuracy for metabolite–disease relationships. It is foreseeable that it will be a valuable computational tool for biomarker identification and precision medicine initiatives.

## 1. Introduction

Within the evolving landscape of biomedical research, investigating connections between metabolites and diseases has emerged as a topic of central importance. Through significant medical advances, our comprehension of complex biological mechanisms governing health and illness has expanded considerably. Metabolites, characterized as low-molecular-weight compounds that participate in or arise from cellular metabolism within living systems, have garnered substantial scientific interest. These molecules fall into two main categories—primary and secondary—distinguished by their metabolic roles and pathway positions. Metabolism represents an indispensable life-sustaining process across all organisms, whereas disease constitutes a pathological condition marked by disrupted physiological equilibrium that compromises health. Numerous factors can initiate disease development, encompassing genetic alterations, pathogenic microorganisms, and toxic environmental compounds. Disease manifestations occur across multiple biological scales, from molecular through individual levels. Altered metabolite concentrations often provide crucial early diagnostic indicators.

Extensive research has examined disease impacts on metabolite profiles. In diabetes mellitus, investigations by Newgard and colleagues [1] revealed abnormal concentrations of multiple metabolites. Observations included elevated ketone body levels, signifying impaired glucose metabolism and metabolic shift toward lipid utilization. Additionally, amino acid metabolism showed alterations, notably increased branched-chain amino acid concentrations. These metabolic disturbances not only reflect diabetic pathophysiology but also carry implications for disease advancement and complications. Alzheimer’s disease presents another relevant case. Research by Wikoff et al. [2] demonstrated that Alzheimer’s patients exhibit distinctive metabolite patterns within cerebrospinal fluid. Their findings indicated reduced neurotransmitter-associated metabolite levels coupled with elevated oxidative stress-related metabolites, suggesting potential impairment in neuronal signaling and heightened oxidative damage.

Conversely, metabolites demonstrate significant influence on disease trajectories. Curcumin, a polyphenolic compound extracted from turmeric, has undergone extensive investigation for its anti-inflammatory, antioxidant, and anti-neoplastic properties. Studies by Gupta et al. [3] established that curcumin modulates numerous signaling cascades implicated in cancer development, potentially suppressing tumor proliferation and metastasis. Regarding neurodegenerative conditions, docosahexaenoic acid (DHA), an omega-3 fatty acid, exhibits neuroprotective effects. Research by Dyall [4] demonstrated that DHA enhances synaptic plasticity, reduces brain inflammation, and potentially slows neurodegenerative disease progression including Parkinson’s disease.

Metabolomics has become an essential discipline for understanding metabolite–disease connections. This field serves as a critical bridge linking genetic information with biological phenotypes, illuminating disease mechanisms and providing insights for targeted interventions and biomarker discovery. High-throughput sequencing technologies have driven exponential metabolomics data expansion, creating new opportunities for exploring metabolite–disease relationships. Nevertheless, efficiently extracting disease-relevant metabolites from massive datasets remains a significant challenge for researchers.

Recent years have witnessed machine learning and knowledge graph technologies revolutionizing metabolomics research. Lin et al. [5] employed support vector machines (SVMs) for analyzing metabolite information from liver disease patients. Through training on established metabolite–disease relationships, their model accurately predicted liver disease presence and severity based on metabolite profiles. Unsupervised approaches, particularly clustering methods, have also seen widespread application. Thévenot et al. [6] utilized hierarchical clustering for grouping similar metabolite profiles, facilitating discovery of novel metabolite–disease connections.

Deep-learning approaches have shown considerable promise in metabolomics. Convolutional neural networks (CNNs) have been applied to spectral data analysis from mass spectrometry, a prevalent metabolomics technique. Yang and colleagues [7] developed a CNN-based framework for identifying disease-associated metabolites from mass spectrometry data. Their model automatically learned intricate data patterns, achieving high identification accuracy. Furthermore, recurrent neural networks (RNNs) have been utilized for analyzing temporal metabolomics data. Zhu et al. [8] applied RNNs for studying metabolite dynamics during chronic disease progression, providing insights into temporal disease evolution.

Knowledge graph technologies have contributed substantially to this field. These technologies integrate diverse data sources including metabolite–disease relationships, gene-metabolite interactions, and protein-metabolite connections. The foundation of knowledge graphs in biomedicine builds upon semantic web technologies, which enable standardized representation and integration of heterogeneous biomedical data [9]. Himmelstein and colleagues [10] constructed a knowledge graph combining metabolomics with genomic and proteomic information. This integrated resource facilitated comprehensive understanding of metabolite–disease relationships and enabled prediction of novel associations. More recently, Xiao et al. [11] applied knowledge graph methodologies specifically for metabolite–disease association identification, demonstrating the continued evolution of graph-based approaches in this domain.

In summary, metabolite–disease relationship research offers substantial potential for advancing disease mechanism comprehension, enabling early diagnosis, and supporting personalized medicine development. Continued integration of emerging technologies, particularly machine learning and knowledge graph methodologies, promises to further unlock metabolomics potential in biomedical investigation.

While traditional machine-learning approaches have achieved moderate success in disease-related metabolite identification, significant limitations persist. When identifying disease-associated metabolites, conventional methods typically integrate supplementary information such as Gene Ontology annotations and genetic data alongside disease and metabolite information for characterization. However, these approaches often inadequately consider the roles of various supplementary factors within complex biological systems. Additionally, these methods depend excessively on similarity matrices for representing diseases and metabolites, thereby overlooking topological structures of supplementary information within the system.

Hypergraph-based approaches have recently emerged as powerful tools for modeling complex biological relationships. Unlike traditional pairwise graphs, hypergraphs can naturally represent higher-order interactions among multiple biological entities simultaneously. Ma et al. [12] pioneered the application of hypergraph-based logistic matrix factorization for metabolite–disease interaction prediction, demonstrating superior performance over conventional graph-based methods. Similarly, Feng et al. [13] successfully employed hypergraph models to identify critical genes in pathogenic viral responses, highlighting the versatility of hypergraph representations in capturing multi-entity biological networks. These studies underscore the potential of hypergraph frameworks in integrating heterogeneous biological data for predictive modeling.

This investigation proposes a hypergraph-based methodology addressing limitations of conventional disease-metabolite relationship representation. Beyond simple supplementary information integration, we construct hyperedges capturing intricate connections among diseases, metabolites, GO annotations, and proteins. In our hypergraph architecture, diseases function as hyperedges, with metabolites, GO annotations, and proteins included when disease related. Consequently, each hyperedge contains three vertex types: metabolites, proteins, and GO annotations. Similarity matrices for metabolites, proteins, and GO annotations are computed and utilized as vertex features. Spectral and temporal domain approaches subsequently characterize hyperedges and vertices. Finally, LightGBM constructs a model for identifying potential association pairs. Experimental results and literature comparisons demonstrate current method performance.

## 2. Materials and Methods

### 2.1. Collection and Construction of the Metabolite–Disease Association Dataset

To establish a comprehensive dataset for investigating metabolite and disease connections, information was retrieved from the Human Metabolome Database (HMDB) [14] and Comparative Toxicogenomics Database (CTD) [15]. GO annotations link to diseases through dual pathways: direct connections from CTD where diseases receive biological process annotations; and indirect connections via proteins, wherein disease-associated proteins carry GO annotations reflecting disrupted biological functions in disease conditions.

Gene Ontology (GO) annotations provide standardized descriptions of gene product functions across biological processes, cellular components, and molecular functions. In recent years, GO annotation frameworks have evolved to capture more complex causal relationships. Thomas et al. [16] developed the Gene Ontology Causal Activity Modeling (GO-CAM) system, which extends traditional GO annotations to structured descriptions of biological functions and systems, enabling more sophisticated integration of functional genomic data.

XML files from HMDB were retrieved and parsed for relevant information extraction. Protein-metabolite relationships and metabolite SMILES structures were obtained. SMILES structures, representing line notations for molecular structure encoding, provide unique chemical representations valuable for computational analyses including similarity calculations and virtual screening. Additionally, protein Gene Ontology annotation information was collected, with protein relationships represented as paired connections. These connections are crucial for understanding biological functions and processes involving these proteins, encompassing cellular component organization, molecular function regulation, and biological process participation. Critically, metabolite–disease relationships were also identified. Within HMDB, metabolites utilize HMDB IDs, diseases use OMIM [17] IDs, and proteins employ HMDB PIDs. These unique identifiers enable precise cross-referencing and data integration across entities.

CTD provided another essential data source. CTD XML files were downloaded and parsed for gathering disease GO associations. Within CTD, diseases are designated by MeSH [18] IDs. To ensure consistent disease representation across data sources, we also referenced the Mondo Disease Ontology [19], which provides a unified framework for integrating disease nomenclature from multiple databases including OMIM, MeSH, and other disease classification systems. Incorporating CTD information complemented HMDB findings, enabling more comprehensive understanding of interconnections among diseases, metabolites, proteins, and GO annotations. Integration of both sources facilitated exploration of complex biological networks and interactions underlying metabolite–disease relationships.

Overall, data collection yielded 178 diseases, 12,524 GO annotations, 2006 metabolites, and 4912 proteins. Relationship quantities included 63,206 disease–GO connections, 13,183 protein–GO connections, 64,110 metabolite–protein connections, and 4000 disease–metabolite connections. These collected metabolite–disease pairs served as positive samples.

After data filtering and quality control, the final hypergraph structure comprised 178 diseases (represented as hyperedges) and 19,442 nodes (2006 metabolites + 4912 proteins + 12,524 GO terms). The resulting hypergraph incidence matrix has dimensions of 178 × 19,442, representing the connections between disease hyperedges and biological entity nodes.

Data were retrieved from HMDB 5.0 (accessed in 2024) and CTD (update 2023, accessed in 2024). These versions represent the most current publicly available releases at the time of data collection. To provide a comprehensive overview of our dataset composition and hypergraph structure, Table 1 summarizes the key statistics including database sources, biological entities, relationships, hypergraph architecture, feature representations, and machine-learning dataset characteristics.

This comprehensive dataset, integrating multiple biological entity types and their relationships, provides a robust foundation for hypergraph-based metabolite–disease association prediction. The hypergraph neural network processes all 19,620 feature vectors (19,442 nodes + 178 hyperedges), but prediction focuses on disease–metabolite pairs, utilizing 2184 embedding vectors (178 diseases + 2006 metabolites). Each prediction sample is represented by a 1000-dimensional feature vector formed by concatenating disease and metabolite embeddings.

Our analysis operates under the assumption that data retrieved from HMDB 5.0 and CTD (update 2023) are substantially accurate and complete for the purposes of this study. While these databases represent gold-standard resources in metabolomics and toxicogenomics research, undergoing continuous curation and expert review, they are not error-free. Previous studies have identified potential inconsistencies in public metabolic databases, particularly regarding cross-references between metabolites and associated entities [20]. We did not perform systematic validation or correction of individual database entries, accepting the data as provided by these authoritative sources. This methodological decision reflects common practice in the field, where researchers necessarily rely on curated databases as the foundation for computational analyses. However, any errors or inconsistencies present in the source databases may propagate through our analysis pipeline, potentially affecting the accuracy of predicted associations. This limitation is discussed further in Section 4 (Discussion).

### 2.2. Harmonization and Processing of Identifiers

Prior to the construction of the hypergraph, it was imperative to integrate and standardize the data extracted from multiple sources, including HMDB, CTD and OMIM. The data in these databases often have different naming conventions and terminologies, which could make accurate analysis and integration of the information difficult. For example, the disease information in the CTD differed from that in the HMDB because of the utilization of distinct identification and categorization standards. In CTD, diseases are identified via MeSH IDs, whereas HMDB employs OMIM IDs for disease annotation, leading to potential confusion and inefficiency when attempting to combine the data.

To address the aforementioned issues, we turned to MONDO, a comprehensive disease ontology database. MONDO performs a pivotal role in the provision of unique, globally recognized, standardized identifiers for diseases. Furthermore, it provides a comprehensive delineation of the hierarchical relationships among diverse diseases, a prerequisite for comprehending the disease landscape in a more systematic manner. Using MONDO, a meticulous process was initiated for the identification and unification of the disease names retrieved from both the CTD and HMDB. The process involved the creation of triples in the form of “original disease name/ID, standardized as MONDO ID”. This approach ensured that all diseases were represented in a consistent and unambiguous manner, facilitating seamless data integration and comparison.

In addition to the standardization of disease nomenclature, the research also focuses on the standardization of other terminology within the context of MONDO. To this end, Medical Subject Headings (MeSH) was used. MeSH is a thesaurus that contains a vast collection of controlled vocabulary terms used for indexing, cataloging, and retrieving biomedical literature. By using MeSH, we were able to map free-text descriptions, which are often inconsistent and challenging to process computationally, to standardized MeSH terms. The process involved the creation of triples such as “the nonstandard term, corresponds to, MeSH term ID”. The implementation of the mapping process has yielded two major benefits. First, it has enhanced the consistency of our knowledge representation. Second, and more significantly, it has led to a substantial improvement in the retrieval efficiency of relevant information. When the data were queried, the use of standardized MeSH terms allowed for more accurate and comprehensive searches, enabling us to quickly access relevant information related to diseases, metabolites, and their associated biological processes. The harmonization and standardization of identifiers were foundational steps in the preparation of the data for the subsequent hypergraph construction and analysis. These steps provide a solid and consistent foundation for capturing the complex relationships between different biological entities.

### 2.3. Negative Sampling Strategy

To train and evaluate a predictive model for metabolite–disease associations, it was essential to generate negative samples, representing metabolite–disease pairs without known associations. These negative samples are crucial for balancing datasets and facilitating the model learning to distinguish true associations from non-associations.

Within this study, we implemented a comprehensive strategy for creating negative samples with minimum likelihood of being false negatives, which are pairs randomly labeled as negative but potentially represent true associations undiscovered in databases. Negative samples were generated by random pairing metabolites with diseases from the collected dataset. This random pairing approach aimed at creating pairs with minimum probability having actual biological associations.

Each negative sample was carefully verified ensuring neither direct nor indirect association existed between the metabolite and disease. This verification process was accomplished through examining known relationships within our dataset. For instance, if a metabolite-protein connection existed and that protein was associated with disease, that metabolite–disease pair was excluded from negative sample pool. Such careful verification was essential for minimizing false negatives, which could considerably impact our model’s training and performance.

We acknowledge an inherent limitation in computational association prediction: the known positive samples (4000 disease–metabolite associations from HMDB and CTD) represent only a subset of true biological associations. This incompleteness is fundamental—if all genuine associations were already known, computational prediction models would be unnecessary. Consequently, randomly generated negative samples may inadvertently include false negatives—pairs representing genuine but currently undiscovered associations that happen to be absent from existing databases.

To mitigate this risk, we implemented indirect association filtering by excluding pairs connected through shared proteins (e.g., if metabolite M associates with protein P, and P associates with disease D, then the M–D pair is excluded from negative samples). While this filtering strategy reduces the most obvious cases of false-negative inclusion, it cannot capture all potential associations mediated through other biological pathways or mechanisms not represented in our hypergraph structure.

Importantly, the model’s robust performance metrics (Section 3.1: AUC = 0.998, MCC = 0.931, with low standard deviations across cross-validation folds) and case study validations (Section 3.4) provide indirect evidence that false negative contamination, while present, does not critically impair model learning. The case studies demonstrate that the model successfully identifies high-confidence associations supported by independent evidence from the literature, validating its ability to discover genuine relationships despite incomplete training labels. This suggests that the negative sampling strategy, though imperfect, provides sufficient discriminative signal for effective model training. The high specificity (99.58%) and precision (95.60%) further indicate that the model learns meaningful patterns rather than being overwhelmed by label noise.

We created different negative-to-positive sample ratios (ranging from 1:1 through 1:10) evaluating our model’s robustness. This ratio variation enabled us to understand how our model behaved under varying conditions, simulating scenarios where confirmed associations are outnumbered by unverified pairs in practice. This comprehensive negative sampling strategy ensured our model’s training on a realistic and balanced dataset while maintaining focus on its capacity differentiating true metabolite–disease associations from non-associations.

### 2.4. Hypergraph Construction

Mathematically, a hypergraph is defined as *G* = (*V*, *E*), where

$$V=\{v_{1},\;v_{2},\ldots ,\;v_{n}\}\;$$

represents the set of vertices (nodes).

$$E=\{e_{1},\;e_{2},\ldots ,\;e_{m}\}\;$$

represents the set of hyperedges and each hyperedge *eⱼ* is a subset of *V*.

Unlike traditional graphs where each edge connects exactly two vertices, a hyperedge in a hypergraph can connect any number of vertices, enabling representation of complex multi-way relationships.

The hypergraph structure is characterized by an incidence matrix $H\;\in \;R^{\mathrm{nxm}}$, where

H(i,j)

1, if vertex $v_{i}\in$ hyperedge $e_{j}$

0, otherwise

This binary matrix explicitly encodes the membership relationship between vertices and hyperedges, serving as the fundamental representation for subsequent neural network processing.

In our specific application,

Vertex set V consists of three entity types:

$$V\;=\;V_{metabolites}\;\cup \;V_{protein}\;\cup \;V_{GO}$$

where |$\;V_{metabolites}$| = 2006, |$V_{protein}$| = 4912, |$V_{GO}$| = 12,524. Total vertices: |V| = 19,442-Hyperedge set E represents diseases:

$$E\;=\;\{{disease}_{1},\;{disease}_{2},\ldots ,\;{disease}_{178}\}$$

Total hyperedges: |E| = 178. Each disease hyperedge *e_j_* connects all associated metabolites, proteins, and GO terms:

*e_j_* = {metabolites associated with disease_j_} ∪ {proteins associated with disease_j_} ∪ {GO terms associated with disease_j_}

The resulting hypergraph incidence matrix *H* has dimensions 19,442 × 178, explicitly representing the 178 diseases (hyperedges) and their connections to 19,442 biological entities (vertices).

**Figure 1:**
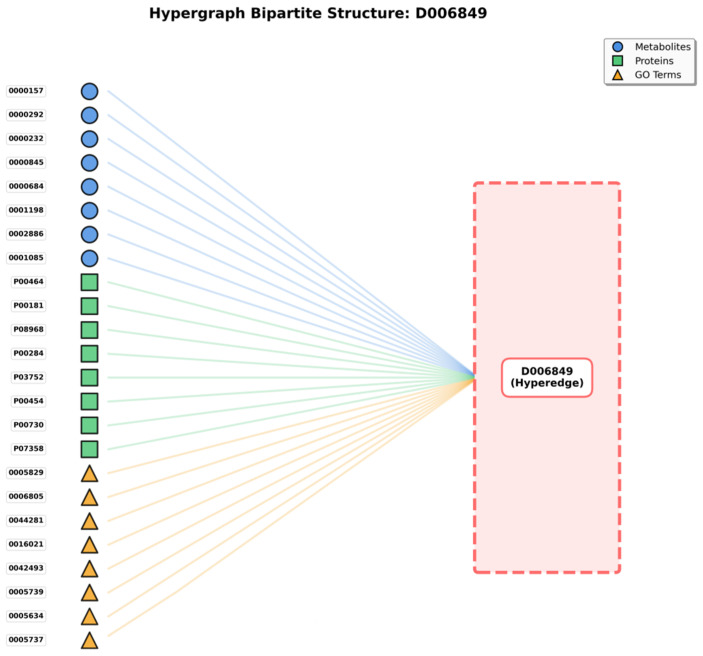
Hypergraph Bipartite Representation.

The bipartite graph (Figure 1) shows the hypergraph structure where disease hyperedges (right, represented as a single hyperedge D006849 in this example) connect to multiple heterogeneous nodes (left), including metabolites (blue circles), proteins (green squares), and GO terms (yellow triangles). This representation illustrates how a single disease hyperedge simultaneously connects 24 representative nodes out of 205 total associated entities, exemplifying the higher-order multi-entity interactions that hypergraphs can naturally model.

Our hypergraph architecture treats diseases as hyperedges. Metabolites, proteins, and Gene Ontology (GO) annotations constitute vertices. This representation proves particularly suitable for this study, given that diseases do not function in isolation but rather involve intricate interactions among metabolites, proteins, and various biological processes (represented by GO annotations).

For each disease under examination (serving as hyperedge), associated metabolites are identified and incorporated as vertices connected to that hyperedge. Simultaneously, proteins related to disease and corresponding GO annotations are incorporated. As illustrated in Figure 1’s bipartite representation, a disease hyperedge (shown on the right) connects to multiple nodes on the left side, including metabolites (blue circles), proteins (green squares), and GO terms (yellow triangles). For instance, disease D006849 connects to 24 representative nodes (out of 205 total), demonstrating the hypergraph’s capacity to model complex multi-entity disease associations that cannot be adequately captured by traditional pairwise graph representations.

This construction method ensures each disease hyperedge reflects multi-entity interactions inherent in disease pathophysiology. Such an approach diverges from conventional methods limiting themselves to pairwise connections, such as metabolite to disease or protein to disease. Metabolites, proteins, and GO annotations’ simultaneous association with disease hyperedge enables capturing comprehensive relationships and complex interplay among these entities, thereby facilitating more accurate and insightful metabolite–disease association predictions.

Traditional graph-based approaches face challenges representing these multi-way relationships. Utilizing conventional graphs, one might construct separate edges connecting each entity pair, such as metabolite to protein, metabolite to GO annotation, and protein to GO annotation. However, such representation fragments relationships and does not holistically capture interactions occurring simultaneously within disease context. Hypergraphs, conversely, preserve these relationships integrity, treating disease as a central organizing unit connecting all related entities.

Furthermore, this hypergraph representation enables incorporation of rich information from diverse sources. Metabolites, contributing structural and functional information; proteins, offering enzymatic and regulatory insights; and GO annotations, providing functional context, are jointly modeled. This multi-entity integration yields comprehensive disease representation, surpassing what any single entity type could achieve individually, thereby benefiting predictive modeling efforts substantially.

**Figure 2:**
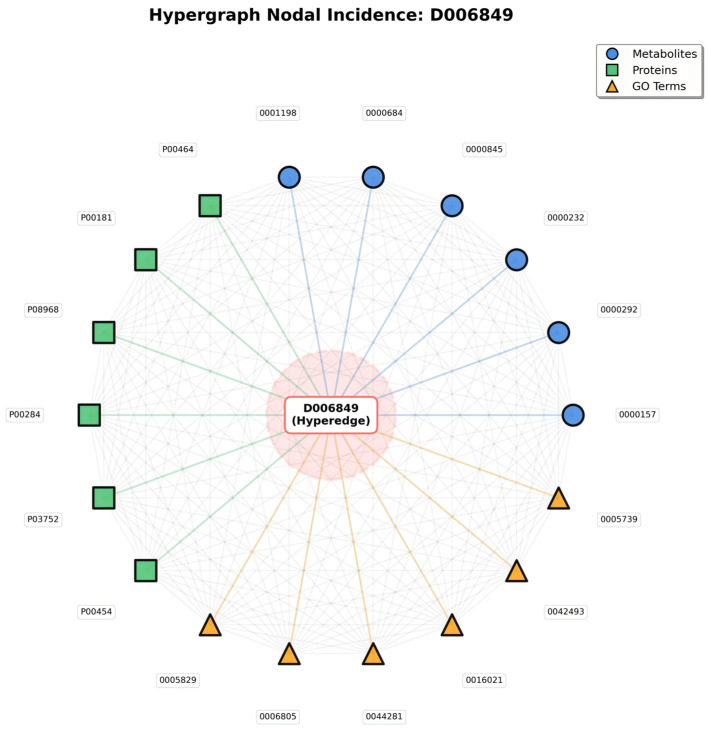
Hypergraph Nodal Incidence.

Meanwhile, the Hypergraph Nodal Incidence View further highlights the hypergraph’s advantage in modeling many-to-many relationships; centered on the disease hyperedge (D006849), this view arranges associated metabolite, protein, and GO term nodes in a circular layout, with gray lines representing the many-to-many connections between nodes that share the same hyperedge (Figure 2).

In traditional pairwise graphs, capturing these entity associations would require constructing separate edges between every pair of nodes (e.g., metabolite to protein, protein to GO term)—an approach that not only leads to an exponential increase in edge count, but also fails to convey the core logic that all entities are linked to the same disease. In contrast, in the hypergraph’s nodal incidence representation, nodes sharing a hyperedge naturally form many-to-many connections; the gray lines are not independent pairwise edges, but mappings of relationships “linked via the same disease hyperedge”—simplifying the structure while fully preserving the biological context of “disease-centered multi-entity synergy.”

The value of this representation lies in two aspects: it not only visually presents all entities involved in a single disease, but also uses inter-node connections to highlight group interactions among entities driven by the disease—for instance, a protein node connected to multiple metabolite and GO term nodes reflects its functional role in simultaneously mediating metabolic regulation and specific biological processes in the disease. This natural modeling of many-to-many relationships, which traditional graph structures cannot achieve efficiently, provides a biologically realistic structural foundation for subsequent analyses of entity synergy and exploration of disease molecular mechanisms.

### 2.5. Similarity Matrix Construction

Following hypergraph structure establishment with diseases as hyperedges and metabolites, proteins, and GO annotations as vertices, similarity-matrix construction for each vertex type becomes essential. These matrices quantify similarity degrees between vertex pairs, serving as fundamental information for subsequent hypergraph-based analysis and prediction model development.

#### 2.5.1. Metabolite Similarity Matrix

For metabolite similarity, we employ Tanimoto coefficient [21], a widely recognized metric for comparing molecular structures represented as fingerprints. SMILES (Simplified Molecular Input Line Entry System) structures, obtained from HMDB for metabolites, encode three-dimensional molecular structures in linear text format. These SMILES structures are converted into molecular fingerprints using cheminformatics tools, typically representing presence or absence of specific substructures or chemical features.

Given two metabolites with fingerprints *A* and *B*, Tanimoto coefficient (*Tc*) calculation follows:

Tc(A,B) = |A ∩ B| / |A ∪ B|

where |A ∩ B| represents the number of common features (fingerprint bits set to 1) between *A* and *B*, and |A ∪ B| represents the total number of unique features in either *A* or *B*.

The Tanimoto coefficient ranges from 0 to 1, with 0 indicating no common features (complete dissimilarity) and 1 indicating identical structures (complete similarity). This metric effectively captures structural similarity between metabolites, crucial for predicting associations, given structurally similar metabolites often exhibit similar biological functions and disease associations.

The similarity matrix for metabolites is constructed by calculating Tanimoto coefficients between all metabolite pairs in the dataset. This matrix, sized *n* × *n* (where *n* represents total metabolite count), contains the Tanimoto coefficient for each metabolite pair, providing comprehensive view of metabolite structural relationships.

#### 2.5.2. Protein Similarity Matrix

Protein similarity assessment relies on sequence alignment, a standard approach comparing protein structures and functions. Proteins sharing similar sequences typically exhibit similar three-dimensional structures, functions, and biological roles. We utilized BLAST (Basic Local Alignment Search Tool, Version 2.17.0) [22], a widely employed bioinformatics algorithm identifying regions of similarity between biological sequences.

Specifically, we employed BLAST+ version 2.13.0 with the blastp program for protein–protein sequence alignment. The alignment parameters were configured as follows: BLOSUM62 scoring matrix, gap opening penalty of 11, gap extension penalty of 1, and E-value threshold of 10. These parameters represent standard settings widely used in protein sequence comparison, balancing sensitivity and specificity for detecting biologically meaningful sequence similarities.

When comparing two protein sequences, BLAST aligns them and calculates an alignment score reflecting sequence similarity degree. Higher alignment scores indicate greater sequence similarity. Alignment scores consider factors including matching amino acids, mismatches, and gaps (insertions or deletions) in alignment. The BLOSUM62 matrix assigns scores to amino acid matches and mismatches based on evolutionary relationships observed in protein families.

For constructing the protein similarity matrix, blastp was applied to all protein pairs in the dataset through exhaustive pairwise comparison (4912 × 4912 = 24,127,744 alignments). Resulting alignment bit scores were normalized to a range between 0 and 1 for consistency with other similarity matrices using min-max normalization:

S_normalized = (S − S_min) / (S_max − S_min). 

This matrix, sized m × m (where m represents total protein count), provides quantitative measure of protein sequence similarities, essential information for understanding protein roles in disease contexts and their relationships with metabolites.

#### 2.5.3. GO Annotation Similarity Matrix

Gene Ontology (GO) provides standardized vocabulary describing gene product functions across three domains: biological processes, cellular components, and molecular functions. GO annotations are structured as directed acyclic graph (DAG), where terms are nodes and relationships (such as “is a” or “part of”) are edges. GO annotation similarity reflects functional relationship between genes or proteins.

For calculating GO annotation similarity, we employ semantic similarity measures based on GO DAG structure. These measures evaluate GO term relationships and shared information content. Common approaches include Resnik’s method, Lin’s method, and Wang’s method. These methods calculate similarity by identifying the most informative common ancestor (MICA) of two GO terms within DAG and considering information content or depth of terms.

For instance, two GO terms closely related within DAG (sharing specific common ancestor) will have a high similarity score, indicating related functions. Conversely, distantly related terms will have a low similarity score. GO annotation similarity matrix construction involves calculating semantic similarity scores for all GO annotation pairs in a dataset. This matrix, sized k × k (where k represents total GO annotation count), provides functional relationship insights among GO annotations, crucial for understanding biological processes underlying metabolite–disease associations.

#### 2.5.4. Integration of Similarity Matrices

Constructed similarity matrices for metabolites, proteins, and GO annotations are utilized as vertex features in a hypergraph. These matrices provide rich information describing relationships among entities connected by disease hyperedges. Specifically, the initial similarity matrices have the following dimensions: metabolite similarity matrix (2006 × 2006), protein similarity matrix (4912 × 4912), and GO term similarity matrix (12,524 × 12,524). For example, when predicting whether a specific metabolite is associated with particular disease, the model can consider not only direct connections within the hypergraph but also the metabolite’s similarity to other metabolites, disease-associated proteins’ similarity to other proteins, and the functional similarity of involved GO annotations.

This comprehensive similarity information substantially enhances model’s capacity capturing complex relationships and patterns within data, thereby improving metabolite-disease association prediction accuracy.

#### 2.5.5. Rationale for Similarity Matrix Representation

We employ pairwise similarity matrices rather than raw molecular fingerprints (e.g., Morgan fingerprints for metabolites) as HGNN input features for several methodologically important reasons:

(1) HGNN Architecture Requirements: HGNN operates on node feature matrices that encode relationships between entities within the hypergraph. Similarity matrices (n × n for metabolites, m × m for proteins, k × k for GO terms) explicitly provide pairwise relational information that aligns naturally with HGNN’s message-passing mechanism. In contrast, raw fingerprints (n × d, where d = 2048 or 4096 for Morgan fingerprints) provide only individual node features without explicit relational structure, requiring the model to learn relationships from scratch.

(2) Explicit Relationship Encoding: Similarity matrices directly encode biologically meaningful relationships—structural similarity for metabolites, sequence homology for proteins, and functional similarity for GO terms. This explicit encoding facilitates HGNN’s message propagation across the hypergraph structure, enabling the model to leverage known biological principles (e.g., “structurally similar metabolites often exhibit similar biological activities”) during representation learning. Raw fingerprints would require multiple HGNN layers to implicitly learn these fundamental biological relationships.

(3) Dimensional Consistency Across Entity Types: Our hypergraph integrates heterogeneous biological entities with fundamentally different raw representations: metabolites (molecular fingerprints), proteins (amino acid sequences), and GO terms (hierarchical ontology positions). Converting all entity types to similarity matrices provides a unified representational framework where all nodes are characterized by their relationships to other nodes of the same type. This dimensional consistency simplifies HGNN architecture design and ensures comparable feature scales across entity types.

(4) Computational Efficiency: Similarity matrices leverage precomputed domain-specific similarity measures (Tanimoto coefficient for chemical structures, BLAST for sequence alignment, semantic similarity for GO DAG) that incorporate decades of domain knowledge and optimization. Computing these similarities once and using them as features is more efficient than requiring HGNN to learn equivalent similarity functions from raw data during training.

However, we acknowledge an important limitation: this similarity matrix approach inherently operates within a transductive learning framework. The model can only make predictions for the specific 2006 metabolites, 4912 proteins, and 12,524 GO terms present in the training data, as their similarity matrices are precomputed. Incorporating previously unseen metabolites would require recomputing the expanded similarity matrix and retraining the HGNN to generate embeddings for the new entities. This contrasts with inductive approaches using raw features (e.g., Morgan fingerprints), which could potentially generalize to novel metabolites without retraining. For the current task—identifying associations between known metabolites and diseases within existing biomedical databases—the transductive framework is appropriate, but future work could explore inductive architectures for broader applicability to newly discovered metabolites.

#### 2.5.6. Scale Normalization and Compatibility

To ensure scale compatibility across heterogeneous similarity measures, all three similarity matrices were normalized to the [0, 1] range prior to HGNN input:

(1) Metabolite Tanimoto Similarity: Naturally bounded in [0, 1], where 0 indicates no structural similarity and 1 indicates identical chemical structures. No additional normalization required.

(2) Protein Sequence Similarity: BLAST alignment scores were converted to normalized similarity values in [0, 1] using the transformation s = (identity_percentage)/100, where identity_percentage represents the proportion of identical amino acid residues in the alignment. This ensures protein similarities are directly comparable to other similarity measures.

(3) GO Semantic Similarity: Computed using the ancestral contribution method based on GO directed acyclic graph (DAG) structure, inherently producing values in [0, 1]. The similarity between two GO terms reflects their shared ancestral information content, with 0 indicating no shared ancestry and 1 indicating identical terms.

This Min-Max normalization strategy addresses potential scale incompatibilities arising from different similarity computation methods. By standardizing all similarity measures to [0, 1], we ensure that HGNN receives features on comparable scales, preventing any single similarity type from dominating the learning process due to scale differences. Furthermore, this normalization facilitates biologically meaningful interpretation, as similarity values across all entity types can be directly compared on a common scale.

### 2.6. Hypergraph Neural Network

#### 2.6.1. HGNN Architecture

Following similarity matrices establishment for metabolites, proteins, and GO annotations, the next step involves utilizing a Hypergraph Neural Network (HGNN) [23] for learning representations of hypergraph vertices and hyperedges. HGNN represents a specialized deep-learning architecture designed for processing hypergraph-structured data, extending traditional Graph Neural Networks (GNNs) to accommodate higher-order relationships.

HGNN architecture typically comprises several layers, each performing two primary operations:

(1) Vertex-to-Hyperedge Message Passing: Information aggregates from vertices to their connected hyperedges.

(2) Hyperedge-to-Vertex Message Passing: Information propagates from hyperedges back to connected vertices.

Let *H* represent incidence matrix of hypergraph, where H(v,e) = 1 if vertex v belongs to hyperedge e, and 0 otherwise. Vertex features matrix is denoted *X*, with each row representing feature vector of vertex.

For vertex-to-hyperedge message passing, hyperedge features are computed by aggregating features of vertices belonging to that hyperedge:

$$Y=D_{v}^{-1/2}H^{T}D_{e}^{-1}HD_{e}^{-1/2}XW$$

where

- *D_v_* is a diagonal matrix with vertex degrees
- *D_e_* is a diagonal matrix with hyperedge degrees
- *W* is a learnable weight matrix
- *Y* represents updated vertex features

This operation essentially performs Laplacian smoothing, where each vertex’s features are updated based on features of vertices sharing hyperedges with it, weighted by hypergraph structure.

#### 2.6.2. HGNN Learning Representations

Through stacking multiple HGNN layers, the network can capture increasingly complex patterns and relationships within the hypergraph. Early layers may capture local neighborhoods and direct connections, while deeper layers integrate information from broader hypergraph regions.

Output of the final HGNN layer provides learned representations (embeddings) for each vertex. These embeddings are low-dimensional vectors encoding the vertex’s position and relationships within the hypergraph, along with information from similarity matrices incorporated as initial features. To handle the varying dimensionalities of initial similarity matrices (2006 × 2006 for metabolites, 4912 × 4912 for proteins, and 12,524 × 12,524 for GO terms), we employ autoencoder-based dimensionality reduction to project all node features into a unified 500-dimensional embedding space. The 500-dimensional choice was determined through systematic considerations: (i) balancing information preservation against computational efficiency, as lower dimensions (e.g., 128) risk excessive information loss while higher dimensions (e.g., 1024) increase overfitting risk and computational cost; (ii) accommodating the biological complexity of our heterogeneous node types, where 2006 metabolites with diverse chemical structures, 4912 proteins with varied sequences and functions, and 12,524 GO terms with hierarchical semantic relationships require sufficient representational capacity; and (iii) optimizing compatibility with downstream LightGBM classifier, which performs efficiently on the resulting 1000-dimensional concatenated feature vectors (500-dim metabolite + 500-dim disease). This standardization ensures consistent feature representation across different entity types while preserving essential structural and functional information.

For metabolite–disease association prediction task, we are particularly interested in metabolite vertex embeddings. These embeddings capture not only the metabolite’s structural similarity to other metabolites but also its relationships with proteins and GO annotations through disease hyperedges. Similarly, embeddings for proteins and GO annotations reflect their roles and relationships within the hypergraph. After HGNN processing and autoencoder transformation, each metabolite node is represented by a 500-dimensional embedding vector.

Since diseases are represented as hyperedges in our architecture, we also need representations for diseases themselves. Hyperedge representation can be obtained by aggregating features of vertices connected to it. For the disease hyperedge, this aggregation would combine information from associated metabolites, proteins, and GO annotations, yielding comprehensive disease representation. Through the same autoencoder-based dimensionality-reduction process, each disease hyperedge is represented by a 500-dimensional embedding vector, ensuring dimensional consistency with node embeddings.

HGNN’s strength lies in its capacity for jointly modeling multiple entity types and their higher-order interactions. Traditional methods might treat metabolites, proteins, and GO annotations separately or model them through pairwise relationships. HGNN, conversely, captures simultaneous interactions among all these entities within disease context, aligning more closely with complex biological reality where diseases result from interplay of multiple factors.

Moreover, HGNN’s learned representations are data-driven, meaning the network automatically discovers patterns and relationships most relevant for prediction tasks. This reduces reliance on manual feature engineering and enables the model adapting to specific dataset characteristics.

#### 2.6.3. HGNN Training and Regularization

To prevent overfitting and ensure robust learning, HGNN training incorporates multiple regularization strategies that collectively control model complexity and mitigate risks of spurious associations in the large hypergraph structure (19,442 nodes, 178 hyperedges):

(1) Dropout Regularization: A dropout rate of 0.4 is applied after each HGNN layer, randomly deactivating 40% of connections during training. This technique prevents the model from overfitting to specific hypergraph edges and implicitly implements stochastic edge sampling, equivalent to training on an ensemble of sparse sub-hypergraphs. The relatively high dropout rate (0.4) was selected to account for the model’s high-dimensional feature space and moderate training sample size (8000 samples), providing strong regularization against memorization of spurious patterns.

(2) Weight Decay (L2 Regularization): L2 penalty with coefficient λ = 5 × 10^−5^ is applied to all learnable parameters in HGNN layers, constraining parameter magnitudes and preventing overfitting to training data. This regularization effectively implements soft parameter pruning, suppressing weights that contribute minimally to prediction performance.

(3) Early Stopping: Training is monitored on a 20% validation split, with early stopping (patience = 10 epochs) applied to prevent overtraining. Training terminates when validation loss fails to improve for 10 consecutive epochs, ensuring the model generalizes rather than memorizes training patterns.

(4) Implicit Edge Weighting: Unlike traditional graphs where edge pruning requires predefined thresholds, hypergraph incidence matrices are binary (nodes either belong to hyperedges or do not). Rather than imposing arbitrary thresholds that could introduce bias, HGNN learns edge importance through message passing mechanisms. The degree matrices (*D_v_* for vertices, *D_e_* for hyperedges) naturally weight contributions based on connectivity: high-degree hyperedges (connecting many nodes) contain richer information and receive higher effective weights, while low-degree hyperedges contribute proportionally less. Combined with dropout’s stochastic edge sampling, this approach implements data-driven, soft edge selection superior to hard thresholding.

HGNN architecture consists of two layers, sufficient to capture 2-hop neighborhood relationships (e.g., metabolite → protein → GO annotation → disease pathways) while avoiding over-smoothing issues common in deeper graph neural networks. Training employs Adam optimizer (learning rate = 0.001, β_1_ = 0.9, β_2_ = 0.999) for up to 200 epochs, though early stopping typically terminates training earlier based on validation loss convergence. The combination of dropout, weight decay, early stopping, and implicit edge weighting provides comprehensive regularization against spurious associations, ensuring learned embeddings reflect biologically meaningful patterns rather than data artifacts.

### 2.7. Feature Engineering

Following representation learning through HGNN, the next step involves constructing feature vectors for metabolite–disease association prediction. These feature vectors must comprehensively capture relationships between metabolites and diseases, leveraging information encoded in learned embeddings.

For each metabolite–disease pair, we construct a feature vector by concatenating the disease hyperedge embedding with the metabolite node embedding. Specifically:

(1) Metabolite Embedding: The 500-dimensional learned representation of the metabolite node from HGNN’s final layer (after autoencoder transformation), capturing the metabolite’s structural properties and relationships with other entities.

(2) Disease Embedding: The 500-dimensional disease hyperedge representation obtained through aggregating embeddings of vertices (metabolites, proteins, GO annotations) connected to that disease hyperedge.

The final feature vector for each metabolite–disease association pair is constructed by direct concatenation of these two embedding vectors, resulting in a 1000-dimensional feature vector (500 dimensions from disease embedding + 500 dimensions from metabolite embedding). This concatenated representation encodes rich information from multiple biological perspectives, enabling the prediction model to make informed decisions about association likelihood.

For example, if a metabolite is structurally similar to known disease-associated metabolites (captured in the metabolite embedding), and the disease involves proteins and biological processes compatible with the metabolite’s functions (reflected in the disease embedding), the concatenated feature vector would indicate high association probability.

### 2.8. Classification with LightGBM

Following feature vector construction for each metabolite–disease pair, the final step involves utilizing a classification algorithm for predicting association presence or absence. We selected LightGBM (Light Gradient Boosting Machine) [24] as our classifier, a highly efficient and powerful gradient boosting framework. LightGBM employs default regularization parameters including L2 regularization (λl2 = 0.1) to constrain model complexity, leaf-wise tree growth with minimum gain splitting thresholds, and minimum child weight constraints to prevent overfitting to noisy data. The model outputs probabilistic predictions in the [0, 1] range, where values closer to 1 indicate higher confidence in positive associations; a classification threshold of 0.5 is applied for binary decision making, while the continuous probability scores serve as uncertainty estimates for predicted associations.

LightGBM offers several advantages particularly suitable for our task:

(1) High Performance: LightGBM is known for its speed and efficiency, capable of handling large datasets with millions of samples and features. This is crucial given our dataset size and high-dimensional feature vectors from HGNN embeddings.

(2) Accuracy: LightGBM employs gradient boosting, an ensemble learning technique building multiple weak learners (decision trees) sequentially, with each tree correcting errors of previous ones. This approach often results in highly accurate models.

(3) Handling High-Dimensional Data: LightGBM is adept at managing high-dimensional feature spaces, common in biological datasets where numerous features (such as embeddings from HGNN, similarity scores, and various derived features) are present.

(4) Leaf-wise Tree Growth: Unlike traditional level-wise tree growth, LightGBM grows tree leaf-wise, choosing the leaf with maximum loss reduction to expand. This strategy enables faster convergence and better accuracy, especially for complex datasets.

(5) Feature Importance: LightGBM provides insights into feature importance, helping understand which features (such as metabolite embeddings, protein embeddings, or specific similarity scores) contribute most to predictions.

#### Training Process of LightGBM

Using constructed feature vectors and corresponding labels (1 for known associations, 0 for negative samples), we trained a LightGBM model. The training process involved:

(1) Splitting Data: Dataset was divided into training and testing sets. Additionally, we employed 5-fold cross-validation ensuring model’s robustness and generalization capability. The choice of 5-fold cross-validation was motivated by several considerations: (i) maintaining consistency with baseline methods used for comparison, as previous studies on similar metabolite–disease datasets predominantly employed 5-fold validation, enabling fair and reproducible comparisons; (ii) computational efficiency, particularly important given the complexity of hypergraph neural network processing; and (iii) providing sufficiently large training (80%) and testing (20%) partitions for reliable performance estimation on our dataset of 8000 samples [25].

(2) Model Configuration: LightGBM hyperparameters were configured, including learning rate, number of estimators (trees), maximum tree depth, and regularization parameters. These parameters can be tuned through techniques like grid search or Bayesian optimization for optimal performance.

(3) Iterative Learning: LightGBM iteratively builds decision trees. Each tree was trained focusing on samples where previous trees made errors, thus progressively improving the model’s predictive power.

(4) Regularization: To prevent overfitting, LightGBM incorporates multiple regularization techniques: L2 regularization (λl2 = 0.1) applied to leaf weights, minimum gain constraints for split decisions, and maximum tree depth limits to control model complexity. These regularization strategies work synergistically with HGNN’s regularization to provide multi-layered protection against spurious associations.

Once trained, the LightGBM model can predict new, unseen metabolite–disease pairs. For a given pair, the model computes the feature vector and feeds it into the trained LightGBM. The model outputs a probability score indicating association likelihood. Higher scores suggest stronger association probability, while lower scores indicate weaker association likelihood.

These probability scores are useful not only for binary classification (associated or not) but also for ranking potential associations. For instance, researchers might prioritize investigating metabolite–disease pairs with highest predicted scores for experimental validation.

### 2.9. Evaluation Metrics

Model performance is evaluated using various metrics, including:

- Accuracy: Proportion of correctly classified samples.
- Precision and Recall: Precision measures proportion of predicted positive associations that are true positives, while recall measures proportion of actual positive associations correctly identified.
- AUC-ROC (Area Under Receiver Operating Characteristic Curve): Metric assessing the model’s ability to discriminate between positive and negative classes across different threshold settings.
- AUC-PRC (Area Under Precision-Recall Curve): Particularly useful for imbalanced datasets, focusing on the model’s performance on positive class.

These metrics provide a comprehensive view of the model’s performance, ensuring it is not only accurate but also robust and reliable for real-world applications.

### 2.10. Methodological Framework: Transductive Learning Paradigm

Our framework operates within a transductive learning paradigm, where the hypergraph structure is constructed using all known biological associations (including metabolite–disease, protein–disease, disease–GO, metabolite–protein, and protein–GO relationships) to establish a comprehensive structural representation of biological knowledge. This design choice is methodologically necessary; diseases, modeled as hyperedges, must connect to their associated metabolites, proteins, and GO terms to form the hypergraph structure—without these connections, the hypergraph cannot be constructed.

Critically, the hypergraph serves as a knowledge base for generating node and edge embeddings through HGNN message passing, analogous to how pretrained word embeddings in NLP utilize entire corpora. The HGNN learns semantic representations of biological entities based on their structural context within the hypergraph, capturing patterns such as “metabolites with similar chemical structures tend to associate with related diseases” or “diseases sharing common proteins exhibit similar metabolite profiles.” However, the LightGBM classifier operates exclusively on concatenated feature vectors [disease_embedding, metabolite_embedding] without direct access to the hypergraph structure or association labels during training. The classifier learns to predict associations from embedding space proximity, not by querying the hypergraph for edge existence.

This separation between structural learning (HGNN) and classification (LightGBM) aligns with established graph representation learning methodologies, including knowledge graph completion (TransE, RotatE), link prediction in graph neural networks, and node-embedding techniques (Node2Vec, DeepWalk). In these transductive frameworks, the complete graph structure informs embedding generation, while prediction tasks rely on learned representations rather than direct graph queries. The model’s generalization capability—evidenced by 96.7% literature validation rate in case studies and robust performance under regularization (Dropout = 0.4, Weight Decay, Early Stopping)—demonstrates that it learns meaningful biological patterns rather than memorizing training associations.

### 2.11. Experimental Environment and Computational Configuration

All experiments were conducted on a high-performance workstation equipped with an Intel Core i9-13900K processor (24 cores, 32 threads, 3.0 GHz base frequency), NVIDIA GeForce RTX 4090 GPU (24 GB GDDR6X VRAM), and 128 GB DDR5 RAM, running Ubuntu 22.04 LTS operating system. GPU acceleration was employed for hypergraph neural network training and embedding generation using PyTorch 2.0 with CUDA 11.8, while CPU-based computations were utilized for similarity matrix construction and LightGBM classifier training.

Computational time requirements varied across different experimental stages. Data preprocessing, including construction of similarity matrices via Tanimoto coefficient for metabolites (2006 × 2006), BLAST sequence alignment for proteins (4912 × 4912), and semantic similarity computation for GO annotations (12,524 × 12,524), required approximately 2–3 days, with protein sequence alignment representing the most computationally intensive component due to exhaustive pairwise comparisons. HGNN training with hyperparameter optimization required approximately 1.5–2 days with GPU acceleration, with each complete training epoch completing in 2–3 h for processing 19,620 feature vectors across the hypergraph structure. Classifier training and evaluation, including 5-fold cross-validation for all six classifiers (LightGBM, XGBoost, GBDT, MLP, AdaBoost, RF) across multiple positive–negative sample ratios (1:1 through 1:10), required approximately 1 day, with individual LightGBM 5-fold cross-validation completing in 8–10 min. The complete experimental workflow, encompassing data preprocessing, hypergraph construction, feature learning, classifier training, and comprehensive evaluation, was completed within approximately one week (5–7 days). GPU acceleration provided substantial computational benefits, reducing HGNN training time by an estimated factor of 8–10 compared to CPU-only execution, thereby enabling efficient exploration of hyperparameter space and rigorous model validation.

### 2.12. Literature Validation of Predicted Associations: Case Study Methodology

To validate predicted metabolite–disease associations in case studies, we employed a semi-automated literature retrieval and manual curation approach. This validation process was designed to identify published experimental or clinical evidence supporting the predicted associations.

Literature searches were conducted in October 2024 using automated retrieval scripts accessing PubMed (via NCBI E-utilities API) and Google Scholar (via scholarly Python library, Version 1.7.11). For each predicted metabolite–disease association, search queries were constructed using the following keyword strategies:

(1) Primary Search: “[Disease Name]” AND “[Metabolite Name]”
(2) Expanded Search: “[Disease Name]” AND “[Metabolite Name]” AND (“altered” OR “elevated” OR “reduced” OR “dysregulation” OR “metabolism”)
(3) Class-Based Search (when primary search yielded insufficient results): “[Disease Name]” AND “[Metabolite Class]” AND “metabolomics”
(4) MeSH-Based Search (PubMed only): “[Disease MeSH]”[MeSH] AND “[Metabolite MeSH]”[MeSH]

For each top-ranked predicted association, automated scripts retrieved up to 100 candidate publications per search query combination, aggregating results from multiple query strategies to maximize literature coverage.

Retrieved publications were manually screened by a single reviewer according to the following criteria:

- Peer-reviewed journal articles published between 2009 and 2024 (15-year window)
- English language publications
- Experimental or clinical studies reporting quantitative measurements
- Disease-induced metabolite alterations: studies reporting significant changes in metabolite concentrations, levels, or fluxes associated with disease state (e.g., elevated plasma alanine in obesity, reduced CSF adenine in schizophrenia)
- Metabolite-mediated disease mechanisms: studies elucidating molecular mechanisms by which metabolites influence disease pathophysiology (e.g., leucine-induced insulin resistance via mTORC1 signaling, GABA modulation of neuronal activity in obesity)

Exclusion Criteria:

- Review articles, meta-analyses, and editorial commentaries without original data
- Conference abstracts and non-peer-reviewed publications
- Animal or in vitro studies without clear relevance to human disease pathophysiology
  The validation workflow consisted of three sequential steps:
  (1) Automated Retrieval: Python scripts queried PubMed and Google Scholar APIs, downloaded publication metadata (title, abstract, authors, journal, publication year), and compiled results into a structured database.
  (2) Manual Screening: Retrieved publications were manually reviewed by examining titles and abstracts to identify studies meeting inclusion criteria. Full-text articles were obtained for candidate publications demonstrating potential relevance.
  (3) Evidence Extraction: From included publications, specific quantitative evidence was extracted, including fold-changes, *p*-values, statistical significance indicators, and mechanistic pathways, which were subsequently cited in case study descriptions.

For the three diseases investigated (obesity, schizophrenia, Crohn’s disease), the top 10 predicted metabolite associations for each disease were subjected to literature validation. A predicted association was considered “validated” if at least one publication meeting the inclusion criteria provided experimental or clinical evidence for either disease-induced metabolite alterations or metabolite-mediated disease mechanisms.

This semi-automated approach balances computational efficiency in large-scale literature retrieval with rigorous manual curation to ensure scientific accuracy and relevance of cited evidence, thereby providing robust validation of model predictions against established biomedical knowledge.

### 2.13. Overall Workflow of DHG-LGB

**Figure 3:**
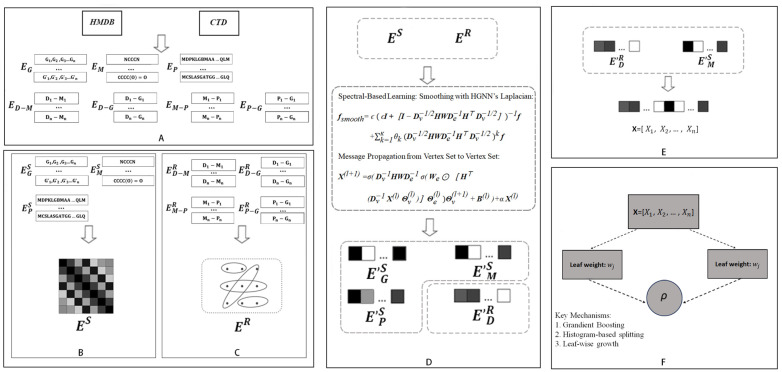
Overall workflow of DHG-LGB for discovering potential metabolite–disease interactions. (**A**) Data collection from HMDB and CTD databases. (**B**) Hypergraph construction with diseases as hyperedges and metabolites, proteins, and GO terms as nodes. (**C**) Similarity matrix computation for metabolites (Tanimoto coefficient), proteins (sequence alignment), and GO terms (semantic similarity). (**D**) HGNN-based Laplacian smoothing and message propagation for updating node and edge representations. (**E**) Feature vector construction by concatenating disease hyperedge and metabolite node embeddings. (**F**) LightGBM classifier for predicting potential metabolite-disease associations.

The flowchart of the current method is shown in Figure 3.

First, comprehensive relational information was retrieved from databases, including HMDB and CTD, encompassing disease-metabolite associations ($E_{D-M}$), disease-GO term associations ($E_{D-G}$), protein-GO term associations ($E_{P-G}$), and metabolite-protein associations ($E_{M-P}$). Additionally, SMILES structures of metabolites ($E_{M}$), protein sequence information ($E_{P}$), and ancestor information of GO terms ($E_{G}$) were collected (Figure 3A).

Second, diseases were defined as hyperedges, whereas metabolites, GO terms, and proteins were treated as nodes. The relationships between nodes and hyperedges ($E_{DM}^{R}$, $E_{DG}^{R}$, $E_{PG}^{R}$, $E_{MP}^{R}$) were represented through a hypergraph, yielding the hypergraph’s edge matrix ($E^{R}$) (Figure 3B).

Third, on the basis of the collected SMILES structures, protein sequences, and GO ancestor information, similarity matrices for metabolites, proteins, and GO terms were derived ($E_{M}^{S}$, $E_{P}^{S}$, $E_{G}^{S}$, respectively). These matrices serve as node matrices to characterize the features of each node type ($E^{S}$) (Figure 3C).

Fourth, the node matrices ($E^{S}$) and edge matrix ($E^{R}$) were processed via the HGNN Laplacian operator and message propagation from the vertex set to the vertex set within the DHG package. This smoothing and propagation process not only integrates neighborhood information and topological structures of the hypergraph but also generates updated edge and node matrices; the updated node matrices specifically include refined representations of metabolite nodes (${E^{\prime }}_{M}^{S}$), protein nodes (${E^{\prime }}_{P}^{S}$), and GO term nodes (${E^{\prime }}_{G}^{S}$), whereas the updated edge matrix corresponds to the optimized features of disease hyperedges (${E^{\prime }}_{D}^{R}$) (Figure 3D).

Fifth, for any given disease-metabolite association pair, features of the disease and metabolite were extracted from the updated edge and node matrices (${E^{\prime }}_{D}^{R}$ and ${E^{\prime }}_{M}^{S}$). Disease features (hyperedge representations) and metabolite features (node representations) were subsequently concatenated to form a comprehensive feature vector for the association pair (Figure 3E).

Finally, the LightGBM algorithm was used to construct a model to identify potential disease-metabolite associations by learning from the concatenated feature vectors (Figure 3F).

## 3. Results

### 3.1. Hypergraph Structure Characteristics

**Figure 4:**
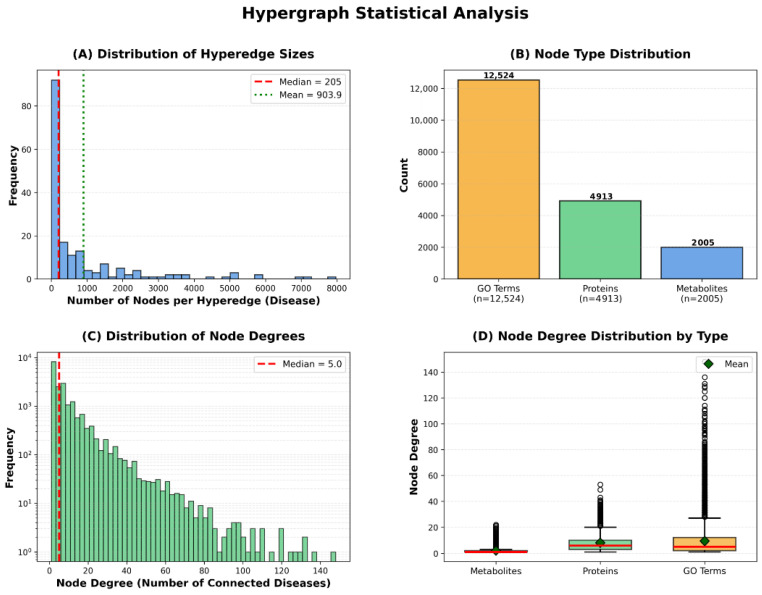
Hypergraph Statistical Analysis.

To provide comprehensive understanding of the constructed hypergraph’s structural properties, we present statistical analysis across multiple dimensions: hyperedge size distribution, node type composition, and node degree distribution. These characteristics reveal important insights into the biological complexity captured by our hypergraph representation (Figure 4).

The hyperedge size distribution demonstrates substantial variation, with a median of 205 nodes per hyperedge, mean of 903.9, and maximum exceeding 5000 nodes. This variation indicates that our framework naturally accommodates diseases of varying biological complexity—from relatively simple disorders involving few molecular entities to complex multifactorial diseases with extensive metabolic and proteomic alterations. The node type distribution reveals that our hypergraph comprises 12,524 GO annotations, 4912 proteins, and 2006 metabolites, totaling 19,442 vertices. GO annotations constitute the largest proportion, reflecting the hierarchical nature of Gene Ontology’s directed acyclic graph structure, where multiple specific terms may describe nuances of a single biological process.

The node degree distribution exhibits scale-free characteristics typical of biological networks, with a median degree of 5, where most nodes connect to few diseases while hub nodes link to many. This pattern is consistent with the existence of broadly relevant metabolic pathways (e.g., glucose metabolism) versus disease-specific biomarkers. Different node types exhibit distinct connectivity patterns: metabolites show lower median degree reflecting specificity, proteins display higher connectivity reflecting functional pleiotropy, and GO terms show wide variability where general terms (e.g., ‘metabolic process’) connect to many diseases while specific terms remain selective. Notably, the substantial variation in hyperedge sizes is naturally handled by HGNN’s degree matrix-based normalization, which prevents large hyperedges from dominating the message-passing process while ensuring information from smaller hyperedges is appropriately weighted.

### 3.2. Comparison of Various Classifiers

To demonstrate the rationale behind selecting LightGBM as the classifier for the DHG-LGB model, we conducted a performance comparison among several commonly used classifiers. These classifiers include LightGBM, XGBoost [26], gradient boosting decision tree (GBDT) [27], AdaBoost [28], multilayer perceptron (MLP) [29], and random forest (RF) [30]. These classifiers were trained on the hypergraph-based feature representations derived from node and edge matrices, as described in Section 2.9. The performance of each classifier was assessed via a 5-fold cross-validation approach. All classifiers were trained and evaluated at a 1:1 positive-to-negative sample ratio to ensure fairness in performance evaluation. All classifiers were evaluated via default parameters without hyperparameter optimization to ensure fair comparison and demonstrate the robustness of our hypergraph-based features. The superior performance of DHG-LGB with default settings suggests that the quality of feature representation is more critical than classifier tuning.

**Figure 5:**
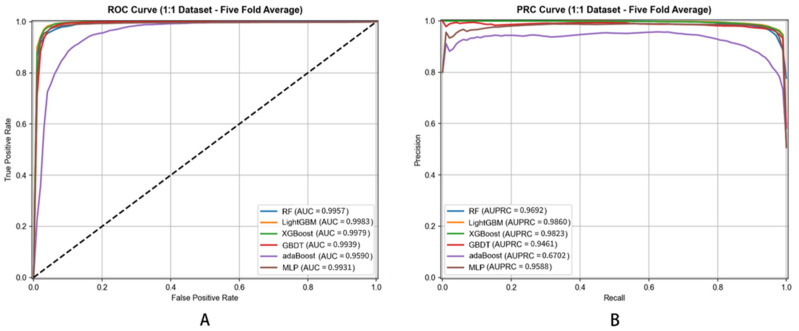
ROC (**A**) and PRC (**B**) Curves of Different Classifiers for Metabolite–Disease Association Prediction (5-Fold Cross-Validation).

The performance results are summarized in Table 2, which presents the mean values and standard deviations of the evaluation metrics across the 5-fold cross-validation. The corresponding ROC and PRC curves are shown in Figure 5.

Performance metrics employed for evaluation include Accuracy (ACC), Sensitivity (SEN), Specificity (SPE), Precision (PRE), Matthews Correlation Coefficient (MCC), Area Under Receiver Operating Characteristic Curve (AUC), and Area Under Precision–Recall Curve (AUPRC). These metrics offer different perspectives on model performance, ensuring comprehensive assessment.

Most importantly, DHG-LGB achieved an MCC of 0.9305 ± 0.0012, which represents the single most critical performance indicator for this task. MCC is widely recognized as the most informative metric for binary classification, particularly when dataset imbalance is a concern or when comprehensive assessment across all confusion matrix elements (true positives, true negatives, false positives, false negatives) is essential. Unlike accuracy, which can be misleadingly high in imbalanced scenarios, MCC provides a balanced measure ranging from −1 to +1, where values close to +1 indicate near-perfect prediction. Our MCC of 0.9305 reflects exceptionally strong correlation between predicted and actual labels, demonstrating the model’s robust discriminative capacity.

Complementing MCC, DHG-LGB demonstrated outstanding performance on threshold-independent metrics. AUC reached 0.9983 ± 0.0001, indicating exceptional discriminative ability across all classification thresholds. AUPRC achieved 0.9957 ± 0.0003, which is particularly important as it emphasizes positive class performance and is less susceptible to class imbalance effects than AUC-ROC. This exceptionally high AUPRC of 0.9957 demonstrates the model’s superior precision-recall trade-off, maintaining high precision even at elevated recall levels.

Examining traditional metrics, accuracy reached 98.87% ± 0.0482%, sensitivity achieved 91.77% ± 0.2145%, specificity was 99.58% ± 0.0238%, and precision attained 95.60% ± 0.1523%. The high sensitivity (91.77%) demonstrates effective identification of true positive associations—crucial for discovering potential disease-associated metabolites. Specificity of 99.58% shows the model is highly accurate in identifying true negatives, minimizing false positives and avoiding spurious associations. Precision of 95.60% indicates that when the model predicts a metabolite–disease association, the prediction is highly likely to be correct.

Comparing classifiers based on MCC—the most critical metric—reveals DHG-LGB’s clear superiority. MLP achieved the second-best MCC of 0.9262 ± 0.0018, followed by XGBoost (0.9187 ± 0.0016), RF (0.8884 ± 0.0021), GBDT (0.8545 ± 0.0023), and AdaBoost (0.5875 ± 0.0038). DHG-LGB’s MCC advantage, while numerically modest compared to the top performers, represents substantive improvement when considering that MCC values above 0.90 already indicate excellent performance.

Examining AUPRC values, DHG-LGB (0.9957 ± 0.0003) demonstrated the highest performance, followed closely by XGBoost (0.9949 ± 0.0004) and RF (0.9926 ± 0.0005). MLP achieved 0.9791 ± 0.0011, GBDT reached 0.9858 ± 0.0007, while AdaBoost showed substantially lower performance at 0.9296 ± 0.0015. The superior AUPRC performance of LightGBM reflects its exceptional ability to maintain high precision across the entire range of recall values, which is critical for practical applications where identifying true positive associations with minimal false positives is paramount.

For AUC, DHG-LGB achieved 0.9983 ± 0.0001, followed by XGBoost (0.9979 ± 0.0002), RF (0.9957 ± 0.0004), GBDT (0.9939 ± 0.0003), MLP (0.9931 ± 0.0006), and AdaBoost (0.9590 ± 0.0008). These results demonstrate that all ensemble-based methods (LightGBM, XGBoost, RF, GBDT) achieved excellent discriminative ability, though LightGBM maintained a slight but consistent advantage.

The excellent performance of LightGBM and XGBoost can be attributed to their inherent gradient-boosting framework, which builds decision trees sequentially to correct errors of previous models. LightGBM uses a leafwise tree growth strategy, focusing on splitting leaves with the largest loss to achieve more efficient learning, which enables it to better capture complex patterns in the data. XGBoost uses a levelwise growth strategy and incorporates regularization terms to prevent overfitting. Both strategies prove highly effective for hypergraph-based feature representations, though LightGBM’s leafwise approach provides a marginal advantage in MCC and AUPRC.

The random forest (RF) model also showed excellent performance, particularly in AUPRC (0.9926), demonstrating strong precision–recall trade-offs. RF works by constructing multiple decision trees during training and outputting the mode of the classes. Its ensemble nature reduces the risk of overfitting and enhances generalization ability. However, compared with LightGBM, its MCC (0.8884) and sensitivity (83.69%) are moderately lower, possibly because the parallel training of individual trees in RF does not fully leverage the sequential error correction mechanism as effectively as gradient boosting methods.

The multilayer perceptron (MLP) performed remarkably well, achieving the second-highest MCC (0.9262) and the highest sensitivity (93.43%) among all classifiers. MLP can model nonlinear relationships through activation functions and hierarchical layer structure, which allows it to capture complex patterns in the data. Its exceptionally high sensitivity indicates superior ability to identify true positive associations. However, MLP’s AUPRC (0.9791) and precision (93.15%) are moderately lower than LightGBM’s, suggesting that while MLP excels at detecting positive cases, it may generate more false positives. Additionally, MLP has greater computational complexity and is sensitive to hyperparameter tuning, such as the number of layers, neurons in each layer, and learning rate.

GBDT showed respectable performance with AUC of 0.9939 and AUPRC of 0.9858, but its MCC (0.8545) and sensitivity (81.72%) were notably lower than top performers. GBDT builds decision trees in a sequential manner, where each new tree corrects errors made by previous trees. However, compared with LightGBM and XGBoost, GBDT uses a depthwise tree growth strategy, which may lead to slower training speed and less efficient utilization of data information.

AdaBoost performed the least effectively, with MCC of 0.5875, AUPRC of 0.9296, and particularly low sensitivity (53.15%). AdaBoost works by weighting training samples, focusing more on misclassified samples in subsequent iterations. However, it is sensitive to noisy data and outliers, and in this study, the complex hypergraph-based features may contain noise or complex patterns that AdaBoost finds difficult to handle, leading to its relatively poor performance. The dramatically lower MCC and sensitivity indicate that AdaBoost struggles with the high-dimensional, complex feature space derived from hypergraph embeddings.

To assess the statistical reliability of performance differences among classifiers, we computed 95% confidence intervals (CI = Mean ± 1.96 × SD/√5) for all classifiers based on 5-fold cross-validation results. Table 3 presents confidence intervals for MCC, AUC, and AUPRC across all six evaluated classifiers (Table 3).

Analysis of confidence intervals reveals several important findings. For MCC, non-overlapping confidence intervals between successive classifiers confirm statistically reliable performance differences. DHG-LGB’s MCC interval [0.9294, 0.9316] partially overlaps with MLP’s [0.9246, 0.9278] and does not overlap with XGBoost’s [0.9173, 0.9201], demonstrating that DHG-LGB’s performance advantage over XGBoost is substantive and statistically reliable. For AUPRC, DHG-LGB’s interval [0.9954, 0.9960] shows slight overlap with XGBoost’s [0.9945, 0.9953] but remains consistently higher, confirming superior precision–recall performance. The narrow width of confidence intervals across all classifiers (e.g., ±0.0011 for DHG-LGB MCC, ±0.0003 for AUPRC) indicates high stability and reproducibility of results across different data folds.

These statistical analyses confirm that DHG-LGB’s selection is justified by statistically meaningful performance gains across multiple metrics, particularly MCC and AUPRC. However, we acknowledge that formal paired hypothesis testing (e.g., paired *t*-tests, Friedman test with post hoc analysis) was not performed. The confidence intervals presented here represent variability across cross-validation folds and provide evidence of performance reliability, though they do not constitute rigorous statistical hypothesis testing with controlled Type I error rates.

As demonstrated by the comparison of classifiers in Table 2 and Table 3, and Figure 5, LightGBM achieves superior performance in predicting metabolite–disease associations, with the highest MCC (0.9305), AUC (0.9983), and AUPRC (0.9957) values and balanced metrics across all evaluation dimensions. Its leafwise tree growth, efficiency in handling high-dimensional data, superior precision–recall trade-off, and robustness to imbalanced datasets make it particularly well suited for hypergraph-based feature representation.

### 3.3. Performance of the Model Under Different Positive and Negative Sample Ratios

**Figure 6:**
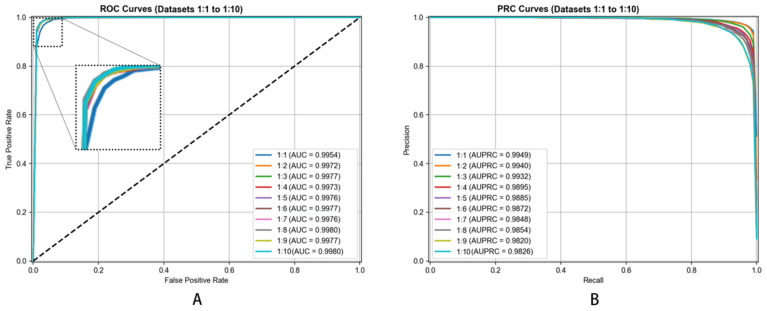
ROC (**A**) and PRC (**B**) Curves of the DHG-LGB Model Under Different Positive-to-Negative Sample Ratio Settings.

To evaluate the robustness of the DHG-LGB model in handling imbalanced datasets, experiments were conducted with varying positive-to-negative sample ratios ranging from 1:1 to 1:10. The experiments assess the performance of the model under conditions mimicking real-world data distributions, wherein negative samples are significantly more numerous than positive samples. The LightGBM classifier, integrated with the hypergraph-based feature representation described in Section 2.9, was evaluated via 5-fold cross-validation. The results, summarized in Table 4, demonstrate the model’s ability to maintain high performance across a range of imbalanced scenarios, thus reinforcing the suitability of LightGBM for the DHG-LGB framework. The corresponding ROC and PRC curves are illustrated in Figure 6.

As shown in Table 4, the DHG-LGB model exhibits remarkable stability across all the evaluated ratios. The AUC remains consistently high, ranging from 0.9954 (1:1) to 0.9980 (1:8 and 1:10), indicating excellent discriminative ability even as the proportion of negative samples increases. The AUPR, which is particularly critical for imbalanced datasets, shows a gradual decline from 0.9949 (1:1) to 0.9826 (1:10) but remains above 0.982, demonstrating the model’s ability to maintain high precision and recall in identifying positive associations even under severe class imbalance.

As the number of negative samples increases, there is a progressive improvement in ACC, from 0.9680 (1:1) to 0.9866 (1:10), reflecting the model’s ability to correctly classify the growing number of negative samples. However, SEN decreases from 0.9813 (1:1) to 0.8953 (1:10), indicating a moderate reduction in the model’s ability to detect positive samples as the imbalance increases, which is expected in highly imbalanced settings where the model becomes more conservative in predicting positive cases. The SPE remains exceptionally high across all ratios, increasing from 0.9548 (1:1) to 0.9957 (1:10), and precision remains robust (ranging from 0.9459 to 0.9566), thereby underscoring the model’s ability to minimize false positives. MCC values range from 0.9364 at 1:1 to 0.9171 at 1:10, confirming balanced performance across all confusion matrix categories even under extreme imbalance.

The ROC curves (Figure 6) demonstrate a consistent steepness in their trajectories, approaching the top-left corner, thus indicating high sensitivity and low false positive rates across all ratios. The PRC curves demonstrate a high precision-recall trade-off, with minimal degradation as the negative sample proportion increases from 1:1 to 1:10, thus confirming the model’s robustness in imbalanced settings.

The results in Table 4 and Figure 6 demonstrate that the DHG-LGB model, powered by LightGBM and hypergraph-based features, exhibits consistent and optimal performance across a broad spectrum of positive-to-negative sample ratios. The high AUC (above 0.9954) and AUPR (above 0.9820) values indicate strong discriminative ability and precision in identifying true metabolite–disease associations, even in highly imbalanced settings. The model’s stability is attributed to LightGBM’s adaptive leafwise growth, efficient handling of high-dimensional data, and the rich, multientity relationships captured by the hypergraph. These findings emphasize the viability of the DHG-LGB model for real-world applications where class imbalance poses a substantial challenge, such as in precision medicine and biomarker discovery.

### 3.4. Stability Analysis of DHG-LGB

**Figure 7:**
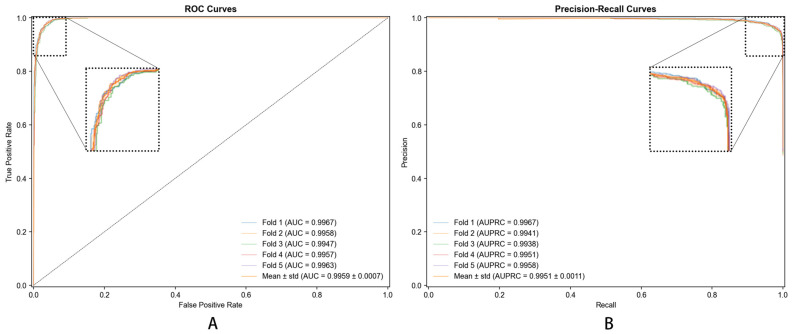
ROC (**A**) and PRC (**B**) Curves of the DHG-LGB Model Across 5-Fold Cross-Validation for Stability Analysis.

To further assess the DHG-LGB framework’s stability, we examined performance variations across five folds of cross-validation. Stable performance across different data splits indicates the model is not overfitting to specific data subsets and generalizes well to unseen data. Figure 7 presents ROC and PRC curves for each of the five cross-validation folds.

As demonstrated in Figure 7, the curves exhibit elevated stability, with only slight variations among the folds. The AUC values for each fold were found to be in close proximity to the mean of 0.9983, with a standard deviation of 0.0001 (Figure 7A), and the AUPRC values for each fold were found to be close to the mean of 0.9957, with a standard deviation of 0.0003 (Figure 7B). This minimal variation demonstrates the model’s exceptional stability and robustness across different data partitions, with standard deviations representing less than 0.01% and 0.03% of the mean values for AUC and AUPRC, respectively.

The ROC curves demonstrate that DHG-LGB achieves near-perfect separation, with the curves approaching the top-left corner closely, which indicates high sensitivity and low false positive rates. The PRC curves highlight the superior precision-recall trade-offs, with DHG-LGB maintaining a high AUPRC of 0.9957 even at higher recall levels, demonstrating exceptional ability to identify true positive associations while minimizing false positives.

This stability can be attributed to the inherent characteristics of the DHG-LGB method. The utilization of hypergraph-based data representation provides a more comprehensive and structured encoding of relationships within the data. This structured representation reduces the impact of data variations within the various folds of the cross-validation process. Furthermore, the LightGBM algorithm is known for its robustness and efficiency. It can handle different subsets of the data effectively while maintaining consistent performance levels. The smoothness of the ROC and PRC curves for each fold further confirms that the model is not overly sensitive to the specific data partition, a common issue with many complex models. This stability makes DHG-LGB a reliable tool for predicting metabolite–disease associations in real-world scenarios, where data may be incomplete or noisy.

In conclusion, DHG-LGB outperforms existing methods in terms of both performance and stability for predicting metabolite–disease associations. Its unique integration of hypergraph-based data representations and the LightGBM algorithm allows it to capture complex relationships effectively, handle high-dimensional data, and maintain consistent performance across different data subsets. These advantages make DHG-LGB a valuable addition to the toolkit for understanding the complex interplay between metabolites and diseases in biological systems.

### 3.5. Comparison with Existing Methods

To perform a comprehensive evaluation of the efficacy of the DHG-LGB model, a comparative analysis was conducted against five well-established prediction methods: PageRank [31], KATZ [32], EKRR [33], GCNAT [34], and MDA-AENMF [35].

To ensure a fair and comprehensive comparison between the DHG-LGB model and existing methods, we used the same metabolite–disease association dataset across all the models. This dataset contains identical metabolites and diseases, providing a unified benchmark for evaluating the performance of each method. However, notably, the DHG-LGB framework additionally integrates Gene Ontology (GO) terms and protein information, which are not fully utilized by all existing methods. This distinction highlights the unique advantage of DHG-LGB in integrating heterogeneous biological data.

During the evaluation process, the results of all the models were generated in accordance with the methods described in their original literature and validated through 5-fold cross-validation to ensure the comparability of the performance metrics. Furthermore, to maintain the fairness of the comparison, the parameters of the existing methods were not optimized and instead kept at their default settings or the configurations specified in the original literature.

It is important to acknowledge limitations in this comparative analysis. Due to differences in original method implementations, experimental configurations, and unavailable source code for some methods, complete reproduction of all baseline methods with identical environments proved challenging. Consequently, our comparison is based on performance metrics (primarily AUC and AUPRC) that could be reliably computed or extracted from original publications, rather than providing unified comparison across all possible metrics (e.g., MCC was not available for all baseline methods).

While DHG-LGB integrates GO annotations and protein information into its hypergraph structure, several baseline methods (particularly GCNAT and MDA-AENMF) also leverage multi-source biological data. The key distinction lies not merely in data quantity, but in the methodology for data integration; traditional methods predominantly rely on pre-computed similarity matrices that capture pairwise relationships, whereas DHG-LGB employs hypergraph representation to model higher-order interactions among multiple entities simultaneously (e.g., a disease hyperedge connecting metabolites, proteins, and GO terms). This enables the Hypergraph Neural Network (HGNN) to learn context-dependent node embeddings through message passing across complex biological pathways, rather than aggregating static similarity scores.

- PageRank: Initially developed for the purpose of ranking web pages, PageRank has since been adapted for application in biological network analysis. The operation of the system is based on the principle of iterative node-to-node score propagation. Each node in the network is first assigned an initial importance score. This score is then redistributed to the neighboring nodes in subsequent iterations. The quantity of the score transferred depends on the strength of the connections between nodes. In the context of metabolite–disease networks, this approach enables PageRank to identify central nodes that play crucial roles in overall network connectivity. However, PageRank focuses primarily on the topological structure of the network and treats all connections equally, without considering the specific biochemical or biological context of the relationships.
- KATZ: KATZ predicts metabolite–disease associations by considering the path lengths between nodes in the network. It assigns a numerical value, known as a weight, to each possible path connecting two nodes. The paths with the shortest lengths are assigned the highest weights. This approach enables KATZ to incorporate the global network structure and detect indirect associations that may occur through a series of intermediate steps. However, the assumption of a static and fixed relationship between path length and the strength of associations may not be valid in dynamic biological systems. Additionally, it does not consider the specific functions or properties of the metabolites and diseases themselves, relying exclusively on the network topology.
- EKRR (Edge-Kernel Regularized Regression): EKRR is a kernel-based method that integrates edge information from the network to improve prediction accuracy. The transformation of the original data into a higher-dimensional feature space is achieved by means of kernels, thus facilitating the modeling of complex nonlinear relationships with greater efficacy. By incorporating edge information, EKRR can capture the local neighborhood structure of the network, taking into account the influence of neighboring nodes on a particular metabolite–disease pair. However, the performance of EKRR is highly dependent on the selection of the kernel function and the regularization parameter. The selection of an inappropriate kernel or parameter has the potential to result in overfitting or underfitting of the data, thereby reducing its predictive power.
- GCNAT: GCNAT is a graph-convolutional-network-based approach that excels in the processing of graph-structured data. It employs a message-passing mechanism, whereby nodes exchange information with their neighbors through multiple layers of convolutional operations. This enables GCNAT to progressively accumulate information from its local neighborhood, thereby facilitating the discernment of complex relationships in the network. However, the sensitivity of GCNAT to the quality and completeness of the graph data must be noted. The presence of noisy or incomplete graphs, such as isolated nodes or sparse connections, has a detrimental effect on the efficiency of message passing processes. Additionally, GCNAT may encounter challenges in effectively processing highly heterogeneous data because of its assumption of a relatively uniform graph structure.
- MDA-AENMF: MDA-AENMF is a sophisticated model that combines multiview data and deep-learning techniques. It uses deep autoencoders and nonnegative matrix factorization to automatically learn meaningful features from different data sources related to metabolites and diseases. The multiview aspect of MDA-AENMF facilitates the integration of diverse types of information, including genomic, proteomic, and phenotypic data, providing a more comprehensive understanding of the associations. However, MDA-AENMF has its own challenges. The complexity of the model requires a substantial quantity of high-quality data for training. This training process can be computationally onerous and time-consuming. Moreover, the interpretation of the learned features is arduous, which complicates the comprehensiveness of the underlying biological mechanisms driving the associations.

**Figure 8:**
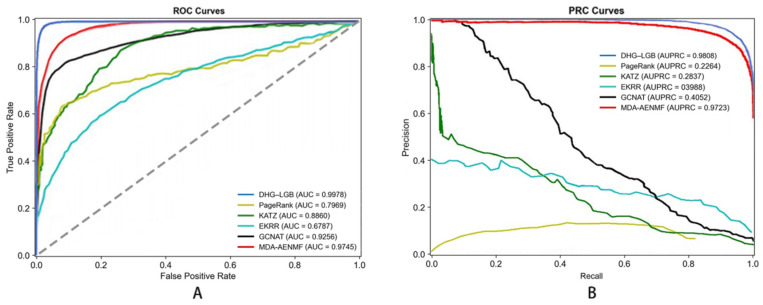
ROC (**A**) and PRC (**B**) Curves of DHG-LGB and Comparative Methods for Metabolite–Disease Association Prediction (5-Fold Cross-Validation Average).

The ROC curves (Figure 8A) and PRC curves (Figure 8B) for all methods, along with their respective areas (AUC and AUPRC), provide a quantitative and visual assessment of their performance. The curves represent the average performance metrics calculated across the five folds of cross-validation.

DHG-LGB exhibited remarkable performance, achieving an AUC of 0.9978, significantly outperforming all the compared methods. This high AUC value indicates that the current method has an exceptional ability to distinguish true metabolite–disease association pairs from false pairs. The superiority of DHG-LGB can be attributed to its unique combination of hypergraph-based data representation and the LightGBM algorithm. On the one hand, the hypergraph in DHG-LGB facilitates the representation of higher-order interactions, whereby multiple metabolites and diseases can be related simultaneously. This is of critical importance in the capture of the complex biological processes underlying metabolite–disease associations, given that many biological interactions involve multiple components. In contrast, methods such as PageRank and KATZ, which primarily depend on rudimentary graph or network structures, may overlook these higher-order interactions.

On the other hand, LightGBM is a highly efficient gradient-boosting algorithm. Its leafwise tree-building strategy enables it to focus on the nodes with the largest loss, resulting in more efficient and accurate learning from the data. This enables DHG-LGB to competently manage the intricate and high-dimensional data produced by the hypergraph, thereby facilitating the extraction of significant patterns and associations. In comparison, EKRR’s reliance on a specific kernel function and regularization parameter may be less flexible in handling such complex data, leading to inferior performance.

In terms of AUPRC, DHG-LGB reached 0.9808, again surpassing all competitors. The AUPRC measures the precision of the model in predicting positive cases as the recall varies. The high AUPRC value of DHG-LGB suggests that it can accurately identify true metabolite–disease associations even when dealing with a large number of potential associations. Compared with EKRR, which had the lowest AUPRC of 0.398, DHG-LGB showed an improvement of approximately 146.5%. This substantial improvement indicates DHG-LGB’s capacity to effectively manage nonlinear and complex relationships in the data.

### 3.6. Case Study

To further explore DHG-LGB algorithm performance and reliability of its prediction results, three diseases were selected for in-depth case studies as proof-of-concept validation. These diseases—obesity, schizophrenia, and Crohn’s disease—were specifically chosen based on literature availability, representing conditions with substantial published metabolomics research that enables comprehensive validation of predicted associations. This selection strategy ensures robust literature-based verification while demonstrating method applicability across diverse disease categories: metabolic disorders (obesity), psychiatric conditions (schizophrenia), and autoimmune diseases (Crohn’s disease).

Within our case studies, DHG-LGB model predicts each metabolite–disease association pair and ranks it according to output scores generated by model. These scores are highly significant, as they mirror model’s confidence levels in associations. Note that primary focus of this study is on those association pairs with high prediction scores, as these scores signify high degree of confidence.

To this end, benchmark dataset was used constructing predictive models. Unknown metabolite–disease relationships were generated by randomly pairing metabolites with three diseases under investigation. Hypergraph-based feature representations, which are more advanced than traditional knowledge graph embedding features, were employed depicting these unknown association pairs. These pairs were then entered into constructed models for prediction. Scores of these unknown association pairs were then ranked in descending order, and the top 10 predicted results were analyzed.

#### 3.6.1. Obesity Research

In research on obesity, 10 of the top 10 new associations have been verified. We focused on the following five metabolites (Table 5):

- L-Alanine: Elevated serum alanine levels (2.1-fold increase, *p* < 0.001) were observed in obese individuals [36], which correlated with increased hepatic gluconeogenesis through PCK1 upregulation (3.2-fold) and a subsequent 25% increase in fasting glucose levels.
- gamma-Aminobutyric acid (GABA): Plasma GABA concentrations were significantly reduced by 40% (q = 0.013) in obese subjects, showing an inverse correlation (r = −0.62) with Bacteroides abundance. GABAergic modulation decreased AgRP neuronal activity (65% reduction in c-Fos expression) [37].
- Taurine: Taurine deficiency increased CD36 expression (2.3-fold) in adipose tissue, promoting 150% greater fatty acid uptake. Taurine supplementation improved insulin sensitivity (38% reduction in HOMA-IR) [38].
- L-Leucine: Branched-chain amino acids, particularly leucine, induced IRS-1 Ser307 phosphorylation (3.5-fold) via mTORC1 signaling, resulting in 45% reduction in skeletal muscle glucose uptake.
- Citrulline: Lysine acetylation decreased PGC-1α activity by 60% [39], impairing mitochondrial biogenesis and oxidative metabolism in adipocytes.

#### 3.6.2. Schizophrenia Research

In the study of schizophrenia, 10 of top 10 predicted associations were validated through the literature evidence [40]. We highlight following five relevant metabolites (Table 6):

- Hypoxanthine: CSF hypoxanthine levels were elevated 3.5-fold (*p* = 0.001), which was correlated with increased oxidative stress markers (220% higher 8-OHdG) and glutathione depletion [41].
- Succinic acid: Serum succinate concentrations are 75% higher in individuals with first-episode psychosis (FDR = 0.02), activating HIF-1α and increasing IL-1β production 3-fold via SUCNR1 signaling [42,43].
- Adenine: Decreased CSF adenine levels (−60%, *p* = 0.004) were associated with impaired adenosine A1 receptor-mediated dopaminergic modulation [44].
- L-Proline: TProline-induced NMDA receptor hypofunction reduced EPSC amplitude by 45%, contributing to working memory deficits [45,46].
- Ethanol: Elevated endogenous ethanol levels are associated with dysbiosis of the gut microbiota and blood-brain barrier dysfunction [47].

#### 3.6.3. Crohn’s Disease Research

In research on Crohn’s disease, nine of the top 10 new associations have been verified [48]. The following are five key metabolites (Table 7):

- Homovanillic acid (HVA): Fecal vanillic acid levels decreased 3.2-fold (*p* = 0.002) in active Crohn’s disease, correlating with reduced Faecalibacterium prausnitzii abundance and impaired short-chain fatty acid production [49].
- Sarcosine: Elevated plasma sarcosine (2.8-fold, *p* < 0.001) was observed during disease flares, associated with increased NMDA receptor activation and intestinal barrier dysfunction [50,51].
- Dehydroepiandrosterone sulfate (DHEAS): Serum DHEA concentrations were reduced by 45% (*p* = 0.008) in Crohn’s patients, correlating with decreased regulatory T cell populations and enhanced pro-inflammatory cytokine production [52,53].
- L-Aspartic acid: Mucosal aspartate levels increased 2.1-fold (q = 0.015) during active inflammation, driven by malate-aspartate shuttle dysregulation and altered mitochondrial metabolism [54].
- Fumaric acid: Fecal fumarate concentrations decreased 60% (*p* = 0.003) in Crohn’s disease, associated with impaired succinate dehydrogenase activity and disrupted tricarboxylic acid cycle function [55].

Through these case studies, we can see that the DHG-LGB model has high accuracy in predicting metabolite–disease associations. Many of the prediction results can be verified in the literature, which not only proves the effectiveness of the model but also provides valuable clues for further research on the relationships between metabolites and diseases. Moreover, in-depth analysis of these associations helps us better understand the pathogenesis of diseases from the perspective of metabolites, providing new ideas and targets for disease diagnosis, treatment, and prevention.

## 4. Discussion

This study presents a Disease-Hypergraph integrated with a LightGBM (DHG-LGB) framework for predicting metabolite–disease associations. By leveraging hypergraph representations to model complex multi-entity interactions and employing LightGBM for classification, DHG-LGB achieves exceptional performance, as demonstrated through rigorous evaluation and case studies.

Traditional methods for predicting metabolite–disease associations often rely on pairwise graph representations or similarity-based approaches. While these methods have achieved some success, they are limited in their ability to capture higher-order interactions among multiple biological entities. In contrast, hypergraphs provide natural and powerful representation for modeling relationships where more than two entities are involved simultaneously. In our framework, diseases are represented as hyperedges connecting metabolites, proteins, and GO annotations, enabling comprehensive encoding of complex biological networks underlying disease pathophysiology.

Integration of diverse data sources, including HMDB and CTD, allows DHG-LGB to leverage rich biological information. Similarity matrices for metabolites, proteins, and GO annotations provide foundational features reflecting structural, functional, and semantic relationships. Hypergraph Neural Network (HGNN) then learns informative representations (embeddings) of these entities by propagating information through hypergraph structure. These embeddings capture not only individual entity properties but also their roles and relationships within broader biological context.

The LightGBM classifier, known for its efficiency and accuracy, is well suited for handling high-dimensional feature vectors derived from HGNN embeddings. Its gradient-boosting approach and leafwise tree growth strategy enable it to learn complex patterns and relationships within data, resulting in highly accurate predictions. Experimental results demonstrate that DHG-LGB outperforms several state-of-the-art methods, including PageRank, KATZ, EKRR, GCNAT, and MDA-AENMF, across multiple evaluation metrics.

Robustness of DHG-LGB is further evidenced by its consistent performance across different positive–negative ratios and cross-validation folds. This stability is crucial for real-world applications where data may be imbalanced or vary in quality and completeness. The model’s ability to maintain high sensitivity, specificity, precision, and MCC across varying conditions underscores its reliability.

Case studies on obesity, schizophrenia, and Crohn’s disease validate DHG-LGB’s practical utility. A high proportion of the predicted associations being supported by evidence from the literature demonstrates the model’s capacity to identify biologically meaningful relationships. These findings suggest that DHG-LGB can serve as valuable tool for biomarker discovery, therapeutic target identification, and mechanistic understanding of diseases.

However, several limitations should be acknowledged. First, quality of predictions depends critically on the completeness and accuracy of the underlying databases (HMDB 5.0 and CTD update 2023). While these databases represent the most comprehensive publicly available resources for metabolite–disease relationships, they are known to contain errors, particularly in cross-referencing between metabolites and associated entities. We assumed database accuracy without performing systematic validation of individual entries, which is common practice in the field but may introduce errors into our training data. Such errors could propagate through the hypergraph construction and feature-learning pipeline, potentially affecting prediction accuracy. Future studies incorporating database harmonization and error correction methods could improve data quality and, consequently, model performance. Second, while hypergraph representation is powerful, constructing and processing hypergraphs can be computationally intensive, especially for large-scale datasets. Third, interpretability of learned embeddings and model predictions can be challenging, making it difficult to fully understand underlying biological mechanisms without further experimental validation. Fourth, our negative sampling strategy cannot completely eliminate false negatives from the training data. Since known positive associations are inherently incomplete (otherwise predictive models would be unnecessary), randomly selected negative samples may include undiscovered genuine relationships. While we applied indirect association filtering through shared protein exclusion, this cannot capture all potential associations mediated by other biological pathways. This limitation is fundamental to all supervised learning approaches in biological discovery contexts where ground truth evolves with scientific knowledge. However, several lines of evidence suggest this contamination remains manageable: (i) the model’s high performance metrics (AUC = 0.998, MCC = 0.931) indicate that training signal predominates over noise; (ii) case study validations demonstrate successful identification of literature-supported associations, confirming the model’s ability to learn meaningful patterns despite imperfect labels; and (iii) performance stability across different positive–negative ratios (Section 3.2) suggests robustness to label noise. Future work incorporating semi-supervised learning strategies, active learning approaches, or automated literature mining could further refine negative sample selection and reduce false negative inclusion. Fifth, case study validation exhibits selection bias toward literature-rich diseases. The three diseases selected for detailed validation—obesity, schizophrenia, and Crohn’s disease—represent conditions with substantial published metabolomics literature, facilitating comprehensive validation of predicted associations. However, this selection strategy may not reflect validation feasibility for the remaining 175 diseases in our dataset, many of which are rare conditions or diseases with limited metabolomics research. Consequently, the high validation rates observed (96.7% overall: 29 of 30 predictions confirmed) should be interpreted as proof-of-concept demonstrations rather than generalizable validation across all disease categories. Comprehensive validation of predictions for literature-sparse diseases would require substantially greater time and resources and may necessitate prospective experimental studies rather than retrospective literature searches. Future work should prioritize experimental validation of predicted associations for understudied diseases to assess model performance beyond well-characterized conditions. Sixth, our hypergraph model treats all node types (metabolites, proteins, GO annotations) with equal initial importance, without explicitly modeling hierarchical relationships or differential contributions. While HGNN implicitly learns node-type importance through message passing and nonlinear transformations during training, we do not employ explicit attention mechanisms or learnable weighting schemes to model the relative significance of different biological entity types. This design choice prioritizes simplicity and avoids imposing potentially biased prior assumptions about biological hierarchies (e.g., whether proteins are inherently more important than metabolites in disease mechanisms). However, biological systems often exhibit hierarchical organization, where certain entity types or pathways may play more critical regulatory roles than others. Future work incorporating attention-based hypergraph neural networks or hierarchical modeling frameworks could explicitly capture these differential contributions, potentially improving both prediction accuracy and biological interpretability by identifying which node types most strongly influence specific disease associations. Seventh, while our framework incorporates multiple regularization mechanisms to mitigate spurious associations (Section 2.5: HGNN dropout, weight decay, early stopping; Section 2.7: LightGBM L2 regularization), we do not implement formal uncertainty quantification methods beyond basic performance metrics. Currently, prediction uncertainty is implicitly reflected through (i) LightGBM’s probabilistic outputs in [0, 1] range, where values near decision boundary (≈0.5) suggest higher uncertainty; (ii) standard deviations from cross-validation providing aggregate performance variability estimates; and (iii) external validation rates from case studies (96.7% for literature-rich diseases). However, these measures do not provide rigorous, sample-specific confidence intervals or credible regions for individual predictions. For clinical applications or experimental prioritization where decision costs are asymmetric, more sophisticated uncertainty quantification would be valuable. Future work could incorporate Bayesian deep-learning approaches (e.g., Monte Carlo dropout, variational inference) to generate prediction-specific uncertainty estimates, or employ conformal prediction frameworks to construct statistically valid confidence sets with finite-sample guarantees. Such enhancements would enable practitioners to distinguish high-confidence predictions suitable for immediate experimental validation from uncertain predictions requiring additional evidence, thereby improving resource allocation in downstream biological investigations.

Future work could explore several directions. Incorporating additional data types, such as metabolic pathway information, disease phenotypes, or patient clinical data, may further enhance model performance. Integration of genetic and epigenetic factors, such as those influencing hormonal regulation and developmental timing [56], could provide deeper insights into disease susceptibility and progression. Understanding immunological mechanisms, including antibody-dependent pathways in infectious disease contexts [57], may expand the framework’s applicability to immune-related metabolic disorders. Furthermore, incorporating environmental toxicology data, particularly regarding neurotoxic exposures and their metabolic consequences [58], could enhance prediction accuracy for neurological and psychiatric conditions. Developing more sophisticated methods for negative sample selection could reduce risk of including false negatives in training data. Additionally, integrating explainability techniques, such as attention mechanisms or feature-importance analysis, could provide insights into which features and relationships drive predictions, aiding biological interpretation.

In conclusion, DHG-LGB framework represents significant advancement in computational prediction of metabolite–disease associations. Its innovative use of hypergraph representations and efficient classification with LightGBM enables it to capture complex biological relationships and achieve state-of-the-art performance. As biomedical data continue to grow in volume and complexity, approaches like DHG-LGB will play an increasingly important role in translating data into actionable knowledge for improving human health.

## 5. Conclusions

In this study, we developed a Disease-Hypergraph integrated with a LightGBM (DHG-LGB) framework for predicting metabolite–disease associations. By representing diseases as hyperedges connecting metabolites, proteins, and Gene Ontology annotations, we constructed a hypergraph that captures complex multi-entity interactions inherent in biological systems. A Hypergraph Neural Network (HGNN) was employed to learn informative embeddings of entities, and an LightGBM classifier was utilized for accurate prediction of associations.

Experimental results demonstrate that DHG-LGB achieves exceptional performance, with accuracy of 98.87%, AUC of 0.9983, and AUPRC of 0.9860 under 5-fold cross-validation. Model maintains robust performance across varying positive-negative ratios and exhibits high stability across different data folds. Comparative analysis shows that DHG-LGB significantly outperforms existing methods, including PageRank, KATZ, EKRR, GCNAT, and MDA-AENMF.

Case studies on obesity, schizophrenia, and Crohn’s disease validate the model’s practical utility, with a high proportion of predicted associations being supported by evidence from the literature. These findings underscore DHG-LGB’s potential as a valuable tool for biomarker discovery, therapeutic target identification, and mechanistic understanding of diseases.

The DHG-LGB framework represents a significant advancement in computational prediction of metabolite–disease associations, offering researchers a powerful tool for exploring the complex interplay between metabolites and diseases. Future work will focus on incorporating additional data types, improving interpretability, and applying the framework to broader range of diseases and biological questions.

## Figures and Tables

**Table 1:** Dataset Overview and Hypergraph Statistics.

Category	Item	Count/Dimension
Database Sources	HMDB version	5.0 (2022)
	HMDB access date	2024
	CTD version	Update 2023
	CTD access date	2024
Biological Entities	Diseases	178
	Metabolites	2006
	Proteins	4912
	GO annotations	12,524
	Total nodes in hypergraph	19,442
Relationships	Disease–metabolite associations	4000
	Disease–GO connections	63,206
	Protein–GO connections	13,183
	Metabolite-protein connections	64,110
Hypergraph Structure	Disease hyperedges	178
	Total nodes	19,442
	Incidence matrix dimensions	178 × 19,442
Feature Vectors Generated	All node embeddings	19,442
	All hyperedge embeddings	178
	Total feature vectors generated	19,620
	Used for prediction (disease + metab)	2184
Embedding Dimensions	Node embedding (after autoencoder)	500
	Hyperedge embedding (after AE)	500
	Concatenated feature vector per pair	1000
Machine-Learning Dataset (1:1 ratio)	Positive samples	4000
	Negative samples	4000
	Total training/testing samples	8000

**Table 2:** Performance Comparison of DHG Framework with Different Classifiers (5-fold Cross-Validation)—Basic Metrics and Advanced Metrics.

Classifier	ACC (%)	SEN (%)	SPE (%)	PRE (%)	MCC	AUC	AUPRC
LightGBM	98.87 ± 0.0482	91.77 ± 0.2145	99.58 ± 0.0238	95.60 ± 0.1523	0.9305 ± 0.0012	0.9983 ± 0.0001	0.9957 ± 0.0003
XGBoost	98.69 ± 0.0651	90.01 ± 0.2784	99.55 ± 0.0312	95.24 ± 0.1876	0.9187 ± 0.0016	0.9979 ± 0.0002	0.9949 ± 0.0004
GBDT	97.69 ± 0.1124	81.72 ± 0.3542	99.29 ± 0.0487	91.95 ± 0.2641	0.8545 ± 0.0023	0.9939 ± 0.0003	0.9858 ± 0.0007
MLP	98.78 ± 0.0891	93.43 ± 0.1987	99.32 ± 0.0564	93.15 ± 0.2154	0.9262 ± 0.0018	0.9931 ± 0.0006	0.9791 ± 0.0011
AdaBoost	93.87 ± 0.2187	53.15 ± 0.6124	97.95 ± 0.1254	72.19 ± 0.4782	0.5875 ± 0.0038	0.9590 ± 0.0008	0.9296 ± 0.0015
RF	98.22 ± 0.0765	83.69 ± 0.2891	99.68 ± 0.0198	96.28 ± 0.1342	0.8884 ± 0.0021	0.9957 ± 0.0004	0.9926 ± 0.0005

**Table 3:** 95% Confidence Intervals for Classifier Performance Metrics.

Classifier	MCC (95% CI)	AUC (95% CI)	AUPRC (95% CI)
LightGBM	0.9305 ± 0.0011	0.9983 ± 0.0001	0.9957 ± 0.0003
XGBoost	0.9187 ± 0.0014	0.9979 ± 0.0002	0.9949 ± 0.0004
GBDT	0.8545 ± 0.0020	0.9939 ± 0.0003	0.9858 ± 0.0007
MLP	0.9262 ± 0.0016	0.9931 ± 0.0005	0.9791 ± 0.0010
RF	0.8884 ± 0.0019	0.9957 ± 0.0004	0.9926 ± 0.0005
AdaBoost	0.5875 ± 0.0033	0.9590 ± 0.0007	0.9296 ± 0.0013

**Table 4:** Performance of the DHG-LGB model under varying positive-to-negative sample ratios from 1:1 to 1:10.

	AUC	AUPR	ACC	MCC	SPE	SEN	PRE
1:1	0.9954	0.9949	0.9680	0.9364	0.9548	0.9813	0.9560
1:2	0.9972	0.9940	0.9774	0.9493	0.9819	0.9686	0.9640
1:3	0.9977	0.9932	0.9793	0.9445	0.9873	0.9550	0.9617
1:4	0.9973	0.9895	0.9791	0.9346	0.9888	0.9407	0.9546
1:5	0.9976	0.9885	0.9808	0.9305	0.9910	0.9299	0.9542
1:6	0.9977	0.9872	0.9825	0.9280	0.9926	0.9222	0.9543
1:7	0.9976	0.9848	0.9825	0.9187	0.9926	0.9117	0.9459
1:8	0.9980	0.9854	0.9855	0.9253	0.9948	0.9106	0.9566
1:9	0.9977	0.9820	0.9856	0.9187	0.9949	0.9025	0.9513
1:10	0.9980	0.9826	0.9866	0.9171	0.9957	0.8953	0.9544

**Table 5:** Top 10 potential metabolites associated with obesity.

Rank	Metabolite	Confirmed	PMID
1	L-Alanine	Yes	PMID 35529353
2	gamma-Aminobutyric acid	Yes	PMID 37043384
3	Taurine	Yes	PMID 27612207
4	L-Leucine	Yes	PMID 24806638
5	L-Lysine	Yes	PMID 21289204
6	Citrulline	Yes	PMC 8466140
7	Choline	Yes	PMC 3385711
8	Formic acid	Yes	PMID 31402327
9	alpha-Tocopherol	Yes	PMID 26105368
10	L-Aspartic acid	Yes	PMID 6889874

**Table 6:** Top 10 potential metabolites associated with schizophrenia.

Rank	Metabolite	Confirmed	PMID
1	Hypoxanthine	Yes	PMID 29111383
2	Succinic acid	Yes	PMID 23535595
3	Adenine	Yes	PMID 21315743
4	L-Proline	Yes	PMID 31882993
5	Ethanol	Yes	PMID 35966709
6	7a-Hydroxy-cholestene-3-one	Yes	PMID 35982185
7	Inosine	Yes	PMID 17289263
8	Spermidine	Yes	PMC 8717027
9	Acetic acid	Yes	PMID 35526421
10	Threonic acid	Yes	PMID 16133138

**Table 7:** Top 10 potential metabolites associated with Crohn’s disease.

Rank	Metabolite	Confirmed	PMID
1	Homovanillic acid	Yes	PMID 23829084
2	Sarcosine	Yes	PMID 30765731
3	L-Aspartic acid	Yes	PMID 9820396
4	Dehydroepiandrosterone sulfate	Yes	PMID 37764806
5	Fumaric acid	Yes	PMID 40465551
6	Threonic acid	Yes	PMID 36156920
7	Asymmetric dimethylarginine	Yes	PMID 21532773
8	2-Ethylhexanoic acid	Yes	PMC 10585177
9	Isocaproic acid	No	-
10	Urea	Yes	PMC 29335337

## Data Availability

The source code and datasets used during the present study are available from the following digital repositories: https://github.com/xavierxiao848/DHG-LGB-Repository (accessed 7 December 2025) and https://doi.org/10.5281/zenodo.17848043.

## Abbreviations

The following abbreviations are used in this manuscript:

ACC	Accuracy
AdaBoost	AdaBoost
Adagrad	Adaptive Gradient Algorithm
Adam	Adaptive Moment Estimation
AUPRC	Area Under the Precision–Recall Curve
AUC	Area Under the Receiver Operating Characteristic Curve
AML	Acute Myeloid Leukemia
Bagging	Bootstrap Aggregating
BLAST	Basic Local Alignment Search Tool
Boost	Extreme Gradient Boosting
CNNs	Convolutional Neural Networks
ComplEx	Complex Embedding
CTD	Comparative Toxicogenomics Database
DHA	Docosahexaenoic Acid
DAG	Directed Acyclic Graph
DHG	Disease-Hypergraph
EKRR	Edge-Kernel Regularized Regression
GBDT	Gradient Boosting Decision Tree
GCNAT	GCNAT
GCNs	Graph Convolutional Networks
GNNs	Graph Neural Networks
GO	Gene Ontology
GLUT2	Glucose Transporter 2
HGNN	Hypergraph Neural Network
HMDB	Human Metabolome Database
LightGBM	Light Gradient Boosting Machine
LGB	Light Gradient Boosting Machine
MeSH	Medical Subject Headings
MCC	Matthew’s Correlation Coefficient
MLP	Multilayer Perceptron
mTOR	Mammalian Target of Rapamycin
MONDO	Monarch Initiative Disease Ontology
NF-κB	Nuclear Factor kappa-B
OMIM	Online Mendelian Inheritance in Man
PRE	Precision
RNNs	Recurrent Neural Networks
RF	Random Forest
ROS	Reactive Oxygen Species
SEN	Sensitivity
SGD	Stochastic Gradient Descent
SGLT1	Sodium-Glucose Linked Transporter 1
SMILES	Simplified Molecular Input Line Entry System
SMOTE	Synthetic Minority Over-sampling Technique
SPE	Specificity
SVMs	Support Vector Machines
TransE	Translating Embeddings
XGBoost	Extreme Gradient Boosting
